# The Patient Protection and Affordable Care Act and Pediatric Medical Clinicians’ Application of Fluoride Varnish

**DOI:** 10.1001/jamanetworkopen.2023.43087

**Published:** 2023-11-14

**Authors:** Tadeja Gracner, Ashley M. Kranz, Kun Li, Andrew W. Dick, Kimberley Geissler

**Affiliations:** 1 RAND Corporation, Santa Monica, California; 2 Center for Economic and Social Research at the University of Southern California, Los Angeles; 3 RAND Corporation, Arlington, Virginia; 4 RAND Corporation, Boston, Massachusetts; 5 Department of Healthcare Delivery and Population Sciences, UMass Chan Medical School-Baystate, Springfield, Massachusetts

## Abstract

**Importance:**

Fluoride varnish reduces children’s tooth decay, yet few clinicians provide it. Most state Medicaid programs have covered this service during medical visits for children aged 1 to 5 years, but private insurers began covering it only in 2015 due to the Patient Protection and Affordable Care Act (ACA) mandate that they cover a set of recommended preventive services without cost-sharing. Evidence on clinicians’ behavior change postmandate is limited.

**Objective:**

To examine monthly changes in fluoride varnish applications among pediatric clinicians following the ACA mandate.

**Design, Setting, and Participants:**

Using all-payer claims data from Massachusetts, this cohort study applied an interrupted time-series approach with linear regression models comparing changes in monthly clinician-level outcomes before and after the mandate. Participants included clinicians who billed at least 5 well-child visits for patients aged 1 to 5 years and were observed at least once premandate. Adjusted for clinician fixed effects, models were assessed overall and separately for clinicians categorized by their monthly share of well-child visits paid by private insurers before the mandate: mostly private (>66% of visits paid by private insurers), mostly public (<33% of visits paid by private insurers), or mixed (33%-66% of visits paid by private insurers) insurance types. Analysis was performed from June 1, 2022, to July 31, 2023.

**Exposure:**

Preenactment and postenactment of the ACA mandate for private insurers to cover fluoride varnish applications without cost-sharing.

**Main Outcomes and Measures:**

Clinician-month measures of whether fluoride varnish was provided during at least 1 well-child visit and the share of such visits, analyzed separately for clinicians who did and did not apply fluoride varnish premandate.

**Results:**

The sample included 2405 clinicians, with 107 841 clinician-months. Premandate, 10.48% of the visits included fluoride varnish applications. Two years postmandate, the likelihood of ever applying fluoride varnish was 13.64 (95% CI, 10.97-16.32) percentage points higher. For clinicians providing fluoride varnish premandate, the share of visits with fluoride varnish increased by 9.22 (95% CI, 5.41-13.02) percentage points. This increase was observed in clinicians who treated children with insurance that was mostly mixed and mostly private; no substantial change was observed among those treating children with mostly public insurance.

**Conclusions and Relevance:**

In this cohort study of pediatric primary care clinicians, an association between the ACA mandate and an increase in fluoride varnish application was observed, especially among clinicians primarily treating privately insured patients and those applying it premandate. However, application remains infrequent, suggesting persistent barriers.

## Key Points

**Question:**

Was the Patient Protection and Affordable Care Act (ACA) mandate that private insurers cover a set of recommended preventive services without cost-sharing associated with clinicians’ application of fluoride varnish during pediatric medical visits?

**Findings:**

In this cohort study of 2405 pediatric primary care clinicians, clinicians were more likely to apply fluoride varnish following the mandate. The largest changes were observed among clinicians who provided at least some fluoride varnish before the mandate and among those who treated largely private or a mix of publicly and privately insured patients.

**Meaning:**

The findings of this study suggest that the ACA preventive services coverage mandate was followed by an increase in pediatric primary care clinicians applying fluoride varnish; however, fluoride varnish provision remained low, suggesting that barriers to its application remain.

## Teaser

This cohort study examines whether changes in the rate of application of fluoride varnish in children occurred following mandate of the Patient Protection and Affordable Care Act.

## Introduction

In 2014, the US Preventive Services Task Force (USPSTF) recommended that medical clinicians apply fluoride varnish to the teeth of all children aged 5 years and younger.^1^ Fluoride varnish applications reduce dental caries^2,3^ and can be cost-saving when applied in medical offices.^4^ Applying fluoride varnish in medical offices improves young children’s access to preventive oral health care because young children are more likely to visit medical offices than dental offices, including 12 recommended well-child visits before the age of 3 years.^5^ Despite recommendations for children to receive fluoride varnish every 3 to 6 months during well-child visits,^5^ rates remain low. Fewer than 10% of well-child visits paid by Medicaid in 22 states during 2006 to 2014 included fluoride varnish applications, and only 4.8% of visits paid by private insurers in 4 states during 2016 to 2018.^6,7^ Additionally, a 2018 national survey of pediatricians found that fewer than 1 in 5 respondents routinely applied or had their staff apply fluoride varnish.^8^

One reason for low clinician engagement is likely the historically confusing payment policy for fluoride varnish. Whereas most state Medicaid programs have paid for fluoride varnish applications during pediatric medical visits since 2008,^9^ many private health insurers only began to pay for it due to the Patient Protection and Affordable Care Act (ACA) preventive services mandate.^9^ As of May 2015, most private health plans were required to cover fluoride varnish applications without cost-sharing due to this mandate, which required coverage of a set of preventive services without cost-sharing for children aged 1 to 5 years.^10^

This study provides new data on whether the enactment of the ACA preventive services mandate was associated with changed clinician delivery of this recommended preventive service. Using the Massachusetts All-Payer Claims Database (APCD), we examined monthly changes in fluoride varnish applications among pediatric primary care clinicians treating children aged 1 to 5 years before and after the ACA mandate. Because this mandate requires coverage without cost-sharing for children with private insurance, but does not change coverage for Medicaid-insured children, we hypothesized that any change in the use of fluoride varnish postmandate would vary based on a clinician’s insurance mix of patients and be greater for clinicians who applied fluoride varnish premandate.

## Methods

This observational cohort study was approved by the RAND Institutional Review Board, and a waiver of informed consent was granted because data were deidentified. This study followed the Strengthening the Reporting of Observational Studies in Epidemiology (STROBE) reporting guideline.^11^

### Study Data and Sample

We used data from the Massachusetts All-Payer Claims Database, version 8.0, for January 1, 2014, through December 31, 2018.^12^ We used information from medical claims for MassHealth (the combined Medicaid and state Children’s Health Insurance Program in Massachusetts) and commercial payers including employer-sponsored insurance, some self-insured employers, health insurance marketplaces, and individually purchased plans. We aggregated these data to the clinician-month level. We limited the sample to clinicians with a primary practice location in Massachusetts with 5 or more well-child visits for children aged 1 to 5 years premandate.^1,5,13^ We excluded those with missing or invalid covariates and required that clinicians were in the data set both premandate and postmandate (eFigure 1 in Supplement 1).

### Exposure Assignment

We defined clinician-months on or after January 1, 2015, as exposed to the ACA mandate that private insurers cover fluoride varnish applications without cost-sharing, and we considered those before January 1, 2015, as unexposed. Although the mandate began May 1, 2015, we chose 2014 as our premandate period since insurers generally operate on the calendar year, as it was also supported in our data.

### Outcomes

Our primary outcome was an indicator for whether fluoride varnish was provided during at least 1 well-child visit for children aged 1 to 5 years in a clinician-month. Our secondary outcome was a monthly share of well-child visits that included fluoride varnish, analyzed separately for clinicians who did and did not apply fluoride varnish premandate in 2014. We modeled these outcomes separately as we expected that the processes that inform a clinician’s decision to initially offer fluoride varnish may differ from the processes that inform a clinician’s decision to offer fluoride varnish during more visits. We used *Current Procedural Terminology* (*CPT*) codes 99382-3 and 99392-3 to identify well-child medical visits for children aged 1 to 5 years and identified fluoride varnish applications on the same service date using *CPT* code 99188 and *Current Dental Terminology* code D1206.

### Clinician Characteristics

We categorized clinicians into 3 groups based on their mean monthly share of well-child visits paid by private insurers at baseline: mostly private (>66% of visits paid by private insurers), mostly public (<33% of visits paid by private insurers), and mixed insurance types (33%-66% of visits paid by private insurers). The baseline period was defined as the time before the ACA mandate (January 1 to December 31, 2014). We also created an indicator for whether clinicians applied fluoride varnish at least once in 2014 (vs never) to examine whether clinicians with existing infrastructure and knowledge needed for its provision responded differently after the mandate than those without it.

### Statistical Analysis

Analysis was performed from June 1, 2022, to July 31, 2023. First, we report unadjusted monthly changes in the share visits during which fluoride varnish was provided 1 year before and up to 2 years after December 2014, overall and by clinician insurance mix (mostly private, mostly public, mixed insurance). We also report unadjusted means (SDs) for continuous variables and proportions for categorical variables at baseline.

Then, we used interrupted time series analyses to assess changes in outcomes before and after December 2014. We estimated linear probability models for binary outcomes and ordinary least-square models for continuous outcomes, all modeled as a linear function of clinician indicators to adjust for unobserved time-invariant factors related to clinicians or baseline differences in their outcomes (ie, clinician fixed effects); county-level measures of dentists per 1000 population and pediatricians and family medicine physicians per 1000 population younger than 18 years^14^; a zip-code-level measure of percentage of the population below 200% of the federal poverty level^15^; and time indicators. We modeled time with a series of monthly indicators, excluding December 2014 as a reference month. These indicators measured mean monthly changes in outcomes relative to the outcomes in December 2014. For the outcome measuring the share of visits with fluoride varnish, we estimated these models separately for individuals applying fluoride varnish premandate and for those who did not.

We estimated models overall and separately based on clinician insurance mix (private, public, or mixed insured), to assess whether postmandate changes varied among subgroups. To determine differences across clinicians, we estimated models with time interacted with insurance-mix indicators and used *t* tests to test for significant differences in responses between the subgroups.

We applied weights to each regression based on the total number of well-child visits for children aged 1 to 5 years per clinician-month to account for variations in visit volume across clinicians. We computed 95% CIs to adjust for clustering within clinicians across monthly observations. We used Stata/MP statistical software, version 17.0 (StataCorp LLC) for analyses. A 2-sided significance threshold was set at *P* < .05. The eMethods in Supplement 1 provides details on estimated models.

We examined the sensitivity of the results to alternative sample specifications. We explored whether results were sensitive to expanding the study sample to include clinicians entering the data set postmandate and to removing the restriction to include those with at least 5 well-child visits premandate. We also reestimated the models by changing the reference month to April 2015 to examine whether changes observed after the official May 2015 enactment date were consistent with our main findings. We calculated the range of predicted probabilities to confirm they lay within the 0 to 1 interval. Additionally, we estimated logistic regression models and compared them with our main findings.

## Results

The study sample included 107 841 clinician-month observations for 2405 unique clinicians (eFigure 1 in Supplement 1). There were 1086 mostly private clinicians, 655 mixed clinicians, and 664 mostly public clinicians (Table). Of these clinicians, 55% were pediatricians, 31% were family practice physicians, and 10% were other clinicians (eg, advanced practice practitioners) (eTable 1 in Supplement 1). The mean (SD) age of clinicians at baseline was 49.1 (11.2) years, and they practiced in areas with 23% of the population below 200% of the poverty level on average. In 2014, we observed a mean of 10.48% clinician-months with any fluoride varnish applications, with the rate observed among mostly public clinicians at 26.86%.

**Table.** Characteristics of Primary Care Pediatric Clinicians Before the Preventive Services Patient Protection and Affordable Care Act Mandate^a^

Characteristic	Mean (SD)^b^			
	Full sample	Clinicians serving mostly publicly insured patients	Clinicians serving both publicly and privately insured patients	Clinicians serving mostly privately insured patients
Clinician-month observations (unique clinicians)	24 072 (2405)	6158 (664)	6617 (655)	11 297 (1086)
Any fluoride varnish provision during well-child visits/mo, No. (%)	24 072 (10.48)	6158 (26.86)	6617 (9.01)	11 297 (2.41)
No. of well-child visits per clinician/mo	12.30 (12.04)	9.44 (11.44)	12.08 (11.36)	13.98 (12.43)
Clinician age, y	49.14 (11.19)	46.60 (11.99)	50.03 (11.05)	50.00 (10.60)
County population <200% of the federal poverty level, %	22.95 (16.77)	35.05 (18.39)	24.42 (15.20)	15.49 (11.96)
Dentists per 1000 county population	0.86 (0.26)	0.86 (0.28)	0.80 (0.28)	0.89 (0.24)

^a^ Descriptive statistics are limited to a sample of clinicians as measured at baseline (ie, January 1 to December 31, 2014).

^b^ Clinicians were categorized based on tercile of well-child visits paid by private insurers: mostly private (>66% of visits paid by private insurers), mostly public (<33% of visits paid by private insurers), and mixed (33%-66% of visits paid by private insurers).

**Figure 1.**
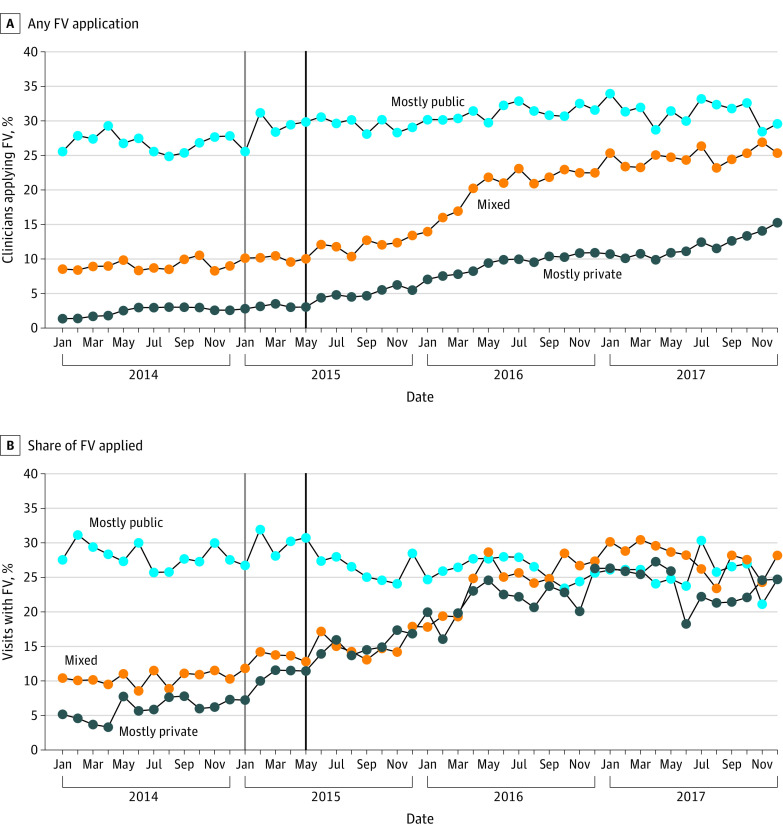
Unadjusted Trends in Monthly Provision of Fluoride Varnish (FV) During Well-Child Visits 1 Year Before and Up to 2 Years After the Implementation of the ACA Mandate A, Unadjusted trends in any FV application per month. B, Unadjusted trends in share of FV applied during well-child visits per month (conditional on applying it at least once in 2014). Clinicians were categorized based on tercile of well-child visits paid by private insurers: mostly private (>66% of visits paid by private insurers), mostly public (<33% of visits paid by private insurers), and mixed (33%-66% of visits paid by private insurers). eFigure 2 in Supplement 1 provides unadjusted trends in the share of FV provided for clinicians providing none premandate. Months before January 2015 (solid light gray vertical line) were defined as the premandate period because, although the mandate began May 1, 2015 (solid dark gray vertical line), insurers typically operate on the calendar year.

Figure 1 depicts unadjusted month-to-month changes in the percentage of clinicians applying any fluoride varnish 1 year before (2014) and after the mandate was enacted (in 2015-2017). Although both figure panels show a visible change in the outcomes soon after January 2015, they also show heterogeneity in clinicians’ responses. Specifically, we observed increases in the percentages of clinicians applying fluoride varnish among those who were categorized as mostly private or mixed premandate in 2014; no similar change was observed among clinicians defined as mostly public premandate. We found similar increases postmandate among clinicians providing no fluoride varnish premandate (eFigure 2 in Supplement 1).

**Figure 2.**
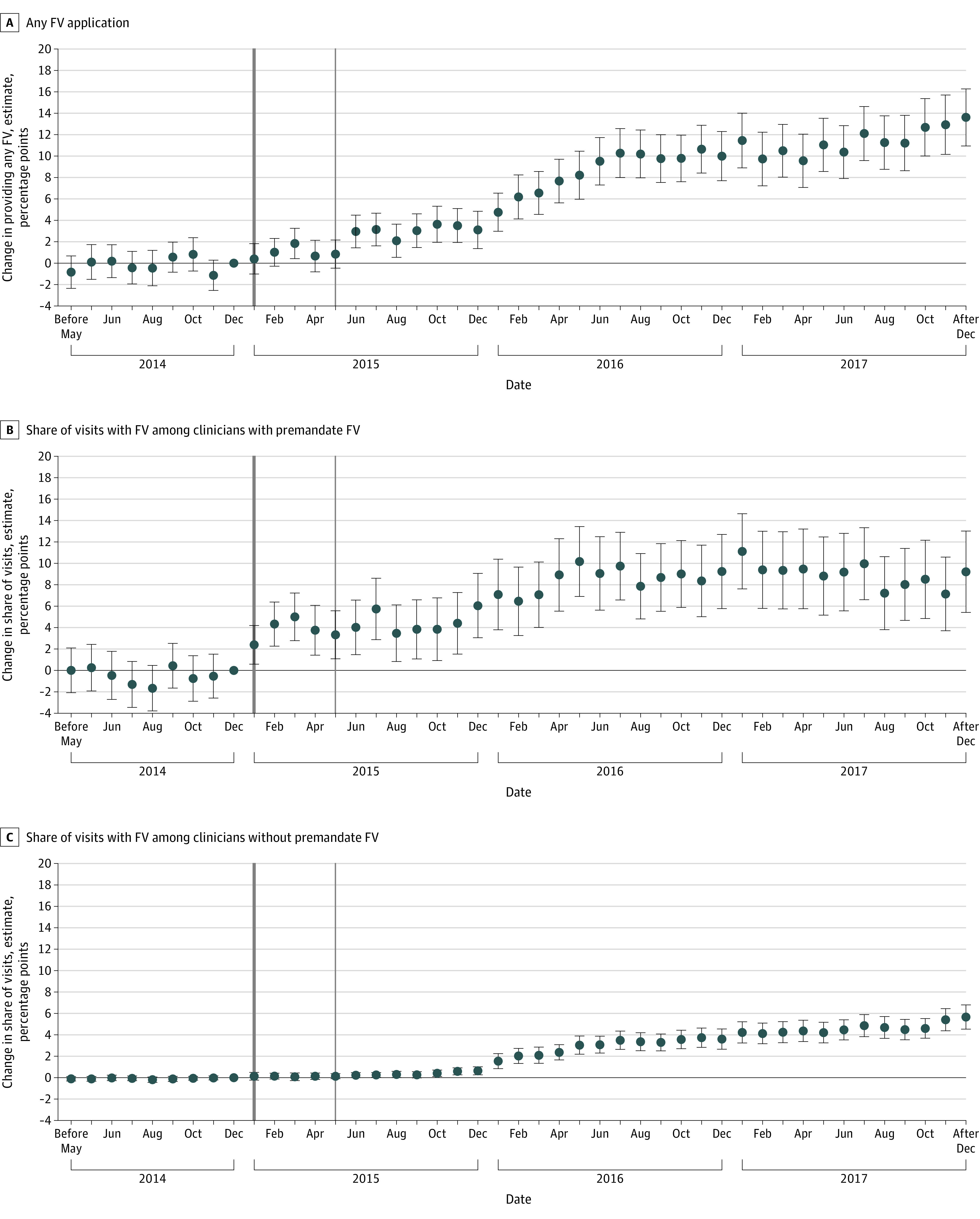
Adjusted Changes in Fluoride Varnish (FV) Provision at Well-Child Visits Relative to December 2014 A, Change in any FV application during a well-child visit per month. B, Change in share of visits with FV per month among clinicians who applied FV premandate. C, Change in share of visits with FV per month among clinicians who did not apply FV premandate. Markers represent the estimated associations at each month relative to December 2014. Months before January 2015 (solid dark gray vertical line) were defined as the premandate period because, although the mandate began May 1, 2015 (solid light gray vertical line), insurers typically operate on the calendar year. Error bars indicate 95% CI. Regression models are provided in eTable 2 in Supplement 1.

Regression-adjusted results show that, compared with December 2014 (ie, the month before we expected to see changes due to the mandate), clinicians were significantly more likely to apply fluoride varnish after the mandate. Compared with December 2014, clinicians were 3.11 (95% CI, 1.37-4.86) percentage points more likely to apply fluoride varnish by December 2015 and 13.64 (95% CI, 10.97-16.32) percentage points by December 2017 (Figure 2A; eTable 2 in Supplement 1). Clinicians also increased the share of visits during which they provided fluoride varnish, with a larger response observed among those who applied it premandate. Compared with December 2014, by December 2017 those clinicians increased the share of visits with fluoride varnish by 9.22 (95% CI, 5.41-13.02) percentage points (Figure 2B; eTable 2 in Supplement 1), compared with a 5.66 (95% CI, 4.53-6.79) percentage points increase among clinicians who did not apply any fluoride varnish in 2014 (Figure 2C; eTable 2 in Supplement 1). We observed an immediate response in behavior in January 2015 among the former and delayed response among the latter.

**Figure 3.**
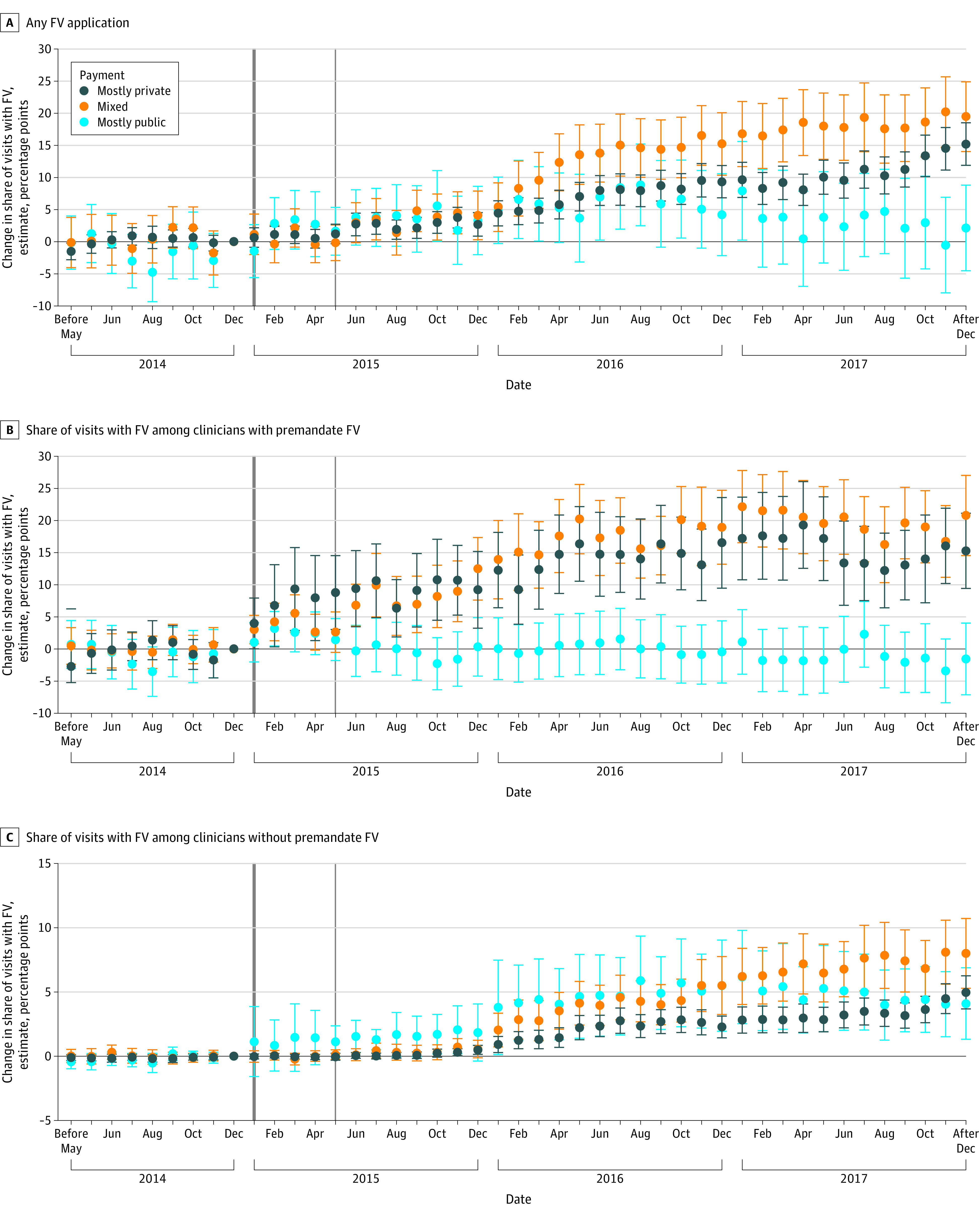
Adjusted Changes in Fluoride Varnish (FV) Applications Relative to December 2014 by Insurance Mix A, Change in any FV application during a well-child visit per month. B, Change in share of visits with FV per month among clinicians who applied FV premandate. C, Change in share of visits with FV per month among clinicians who did not apply FV premandate. Markers represent the estimated associations with corresponding 95% CIs at each month relative to December 2014. Clinicians were categorized based on tercile of well-child visits paid by private insurers: mostly private (>66% of visits paid by private insurers), mostly public (<33% of visits paid by private insurers), and mixed (33%-66% of visits paid by private insurers). Months before January 2015 (solid dark gray vertical line) were defined as the premandate period because, although the mandate began May 1, 2015 (solid light gray vertical line), insurers typically operate on the calendar year. Error bars indicate 95% CI. Regression models are provided in eTables 3 to 5 in Supplement 1.

Increases in any fluoride varnish applications were seen primarily in changes in behavior from mixed clinicians and mostly private clinicians. Compared with premandate, by December 2017, mixed clinicians were (1) 9.47 (95% CI, 14.02-24.91) percentage points more likely to apply any fluoride varnish (Figure 3A; eTable 3 in Supplement 1), (2) increased their share of visits with fluoride varnish applications by 20.78 (95% CI, 14.55-27.01) percentage points among those who provided fluoride varnish in 2014 (Figure 3B; eTable 4 in Supplement 1), and (3) increased their share of visits with fluoride varnish applications by 8.01 (95% CI, 5.29-10.72) percentage points among those who did not apply fluoride varnish in 2014 (Figure 3C; eTable 5 in Supplement 1). Among clinicians who provided at least some fluoride varnish in 2014, a significant change after December 2014 was also observed among mostly private clinicians in fluoride varnish applications, but no significant change was detected among mostly public clinicians compared with premandate (estimate details provided in eTable 4 in Supplement 1). Both mostly mixed and mostly public clinicians who did not apply fluoride varnish in 2014 increased their fluoride varnish provision postmandate (Figure 3C).

Changes in the share of visits with fluoride varnish among those who applied fluoride varnish in 2014 occurred soon after December 2014 for clinicians providing fluoride varnish premandate (Figure 3B) and with delay (especially after May 2015) for those who did not (Figure 3C). Our main findings were consistent using April 2015 as the reference month, although slightly smaller, as expected due to the anticipation effect (eFigure 3 in Supplement 1). The findings were unaffected by expanding the sample to include clinicians entering the data set postmandate, removing the restrictions to include those with at least 5 well-child visits premandate (eFigure 5 and eFigure 6 in Supplement 1) or alternative specifications (ie, estimating logistic regression and excluding clinician fixed effects, eFigure 4 in Supplement 1). Modeled probabilities using linear probability models with fixed effects were between 0 and 1.

## Discussion

To our knowledge, this study is the first to observe that the ACA mandate for private insurers to cover evidence-based preventive services without cost-sharing was associated with an increase in pediatric primary care clinicians applying fluoride varnish during well-child medical visits. In line with prior studies reporting that clinician provision of preventive services, including for fluoride varnish, varies across clinician characteristics,^16,17,18^ training,^19,20^ and patient panel characteristics,^21,22^ we observed that this increase was the largest among clinicians treating a mix of publicly and privately insured patients premandate in 2014.

We found that clinicians treating a mix of publicly and privately insured patients were more likely than clinicians treating mostly private patients to apply any fluoride varnish postmandate. This may be because these clinicians already had infrastructure and familiarity with this service; the percentage of clinician-months that ever applied fluoride varnish premandate was higher among clinicians treating patients with both public and private insurance (9.01%) than clinicians treating mostly privately insured patients (2.41%). This rationale is also supported by our finding that clinicians who applied fluoride varnish before the mandate increased the share of visits with fluoride varnish provision postmandate the most. The increase is largely associated with clinicians with a mixed or privately insured patient base; as expected, those already treating largely publicly insured patients premandate did not respond to this mandate.

Our results showing lower-than-recommended rates of fluoride varnish delivery align with prior research that has documented barriers to the initial adoption of delivering fluoride varnish, both real and perceived.^8,23,24,25^ Despite being a recommended part of medical visits,^1,5,13^ in a 2018 survey of pediatricians, only 54% of respondents indicated that they should apply fluoride varnish.^8^ Clinicians have reported lack of training in oral health, limited time, and challenges with billing as barriers specific to fluoride varnish delivery. Strategies recommended to overcome these barriers include providing training to all practice staff, identifying a staff member to champion this service, and having clear roles about who is responsible for ordering supplies and applying fluoride varnish.^8,24,26^ Additionally, prior research on preventive services has found that clinician recommendation is one of the strongest predictors of patient receipt of these services in adult populations.^27,28,29,30^ This has also been shown in pediatric populations, especially related to routine vaccines. A study by Kempe and colleagues^31^ found that 91% of parents agree with the statement, “I do what my child’s health care provider recommends about vaccines,” suggesting that increasing the number of clinicians offering fluoride varnish is an important way to increase the number of children receiving this service.

Research has shown that payment policy can be a powerful incentive for increasing rates of evidence-based care, including preventive services more generally^32,33^ and fluoride varnish in the medical office specifically.^7^ Prices paid by private insurers closely followed Medicaid rates.^34^ Like many other preventive services, this is a payment additional to that of the well-child visit and so future research might further examine the role of negotiated prices in clinician provision of fluoride varnish.

Conflicting guidance on who should receive fluoride varnish may lead clinicians to apply fluoride varnish infrequently to the teeth of children with private insurance, explaining the smaller and delayed change observed among clinicians who did not apply fluoride varnish before the mandate and who mostly treat children with private insurance. While the USPSTF encourages fluoride varnish applications during well-child visits for all children younger than 6 years with teeth,^1^ updated to children younger than 5 years as of 2021,^13^ some guidelines suggest it is only indicated for children without a usual source of dental care or at high risk of dental caries^35^—characteristics less common among children with private insurance and from higher income families.^36,37^ Previous efforts to increase young children’s receipt of fluoride varnish in medical settings have primarily focused on children with public insurance,^26,38^ suggesting clinicians who treat children with public insurance may be more connected to resources to help them integrate this service into their clinical practice.

Changing guidance about eligibility for screenings and preventive services, including guidance from the USPSTF and others that influence insurance reimbursement, is common.^19,39,40,41^ Our findings are relevant to health care services for which insurance payment and patient cost-sharing is influenced by guidelines. While fluoride varnish application as a preventive service may face higher barriers to implementation than some services due to the need for additional physical resources and mixed support from pediatricians,^8^ our results have implications for understanding clinician response to policy changes. The preventive services mandate is subject to legal challenges that may substantially and differentially impact insurance coverage regulations for preventive services both across and within states.^42,43^

### Limitations

This study has limitations. First, our results may not be nationally generalizable. However, a detailed analysis of clinician behavior over time among private and publicly insured patients allows for the unique analysis of the association between this ACA mandate across a statewide sample of clinicians. Second, our results are robust to alternative specifications, but this is not a randomized trial, so results are not causal. Third, we examined fluoride varnish applications during well-child visits. Although this is when fluoride varnish applications are recommended to occur,^5^ we may undercount fluoride varnish applications if some clinicians routinely provide fluoride varnish during other types of visits. Fourth, although we captured a large share of pediatric primary care clinicians, our findings may be not generalizable to all clinicians practicing in Massachusetts due to our sample restrictions and data limitations. Private self-insured plans were severely reduced starting in 2016 due to a Supreme Court ruling.^12^ Our findings, robust to keeping or relaxing the restriction that clinicians be observed premandate and postmandate, minimize this concern.

## Conclusions

In this cohort study of pediatric primary care clinicians, we observed an increase in fluoride varnish applications following the ACA mandate for private insurers to cover evidence-based preventive services without cost-sharing. The largest increases were among clinicians serving a mix of publicly and privately insured patients and mostly privately insured patients who had provided some fluoride varnish premandate. Future research should examine whether these rates continue to increase or plateau, and whether interventions can increase the number of clinicians applying fluoride varnish.

## Notes

Supplement 1. **eMethods.** Detailed Methods **eFigure 1.** Analytic Sample Construction **eFigure 2.** Unadjusted Changes in Fluoride Varnish Provision at Well-Child Visits Before and After December 2014 for Clinicians Providing no Fluoride Varnish Pre-Mandate **eFigure 3.** Adjusted Changes in FV Applications During Visits Before and After April 2015 **eFigure 4.** Adjusted Changes in Fluoride Varnish Applications at Well-Child Visits Relative to December 2014 Using Alternative Specifications **eFigure 5.** Adjusted Changes in FV Applications During Visits Before and After December 2014, Sensitivity Analysis Allowing Clinicians to Enter the Dataset Post-Mandate **eFigure 6.** Adjusted Changes in FV Applications During Visits Before and After December 2014, Sensitivity Analysis That Removed the Restriction to Include Only Clinicians With at Least 5 Well-Child Visits Pre-Mandate **eTable 1.** Taxonomies of Clinicians in Study Sample **eTable 2.** Regression Model Results That Correspond to Figure 2 **eTable 3.** Regression Model Results That Correspond to Figure 3A **eTable 4.** Regression Model Results That Correspond to Figure 3B **eTable 5.** Regression Model Results That Correspond to Figure 3C

Supplement 2.
Data Sharing Statement
