# Supplementary material for: The Patient Protection and Affordable Care Act and Pediatric Medical Clinicians’ Application of Fluoride Varnish
Source: JAMA Netw Open. 2023 Nov 14;6(11):e2343087. doi: 10.1001/jamanetworkopen.2023.43087 (PMC10646725; doi:10.1001/jamanetworkopen.2023.43087)
Supplement: Supplement 1. — eMethods. Detailed Methods eFigure 1. Analytic Sample Construction eFigure 2. Unadjusted Changes in Fluoride Varnish Provision at Well-Child Visits Before and After December 2014 for Clinicians Providing no Fluoride Varnish Pre-Mandate eFigure 3. Adjusted Changes in FV Applications During Visits Before and After April 2015 eFigure 4. Adjusted Changes in Fluoride Varnish Applications at Well-Child Visits Relative to December 2014 Using Alternative Specifications eFigure 5. Adjusted Changes in FV Applications During Visits Before and After December 2014, Sensitivity Analysis Allowing Clinicians to Enter the Dataset Post-Mandate eFigure 6. Adjusted Changes in FV Applications During Visits Before and After December 2014, Sensitivity Analysis That Removed the Restriction to Include Only Clinicians With at Least 5 Well-Child Visits Pre-Mandate eTable 1. Taxonomies of Clinicians in Study Sample eTable 2. Regression Model Results That Correspond to Figure 2 eTable 3. Regression Model Results That Correspond to Figure 3A eTable 4. Regression Model Results That Correspond to Figure 3B eTable 5. Regression Model Results That Correspond to Figure 3C [file jamanetwopen-e2343087-s001.pdf]

Supplementary Online Content

Gracner T, Kranz AM, Li K, Dick AW, Geissler K. The Patient Protection and Affordable Care Act and pediatric medical clinicians' application of fluoride varnish. *JAMA Netw Open*. 2023;6(11):e2343087. doi:10.1001/jamanetworkopen.2023.43087:

eMethods. Detailed Methods

eFigure 1. Analytic Sample Construction

eFigure 2. Unadjusted Changes in Fluoride Varnish Provision at Well-Child Visits Before and After December 2014 for Clinicians Providing no Fluoride Varnish Pre-Mandate

eFigure 3. Adjusted Changes in FV Applications During Visits Before and After April 2015

eFigure 4. Adjusted Changes in Fluoride Varnish Applications at Well-Child Visits Relative to December 2014 Using Alternative Specifications

eFigure 5. Adjusted Changes in FV Applications During Visits Before and After December 2014, Sensitivity Analysis Allowing Clinicians to Enter the Dataset Post-Mandate

eFigure 6. Adjusted Changes in FV Applications During Visits Before and After December 2014, Sensitivity Analysis That Removed the Restriction to Include Only Clinicians With at Least 5 Well-Child Visits Pre-Mandate

eTable 1. Taxonomies of Clinicians in Study Sample

eTable 2. Regression Model Results That Correspond to Figure 2

eTable 3. Regression Model Results That Correspond to Figure 3A

eTable 4. Regression Model Results That Correspond to Figure 3B

eTable 5. Regression Model Results That Correspond to Figure 3C

This supplementary material has been provided by the authors to give readers additional information about their work.

© 2023 Gracner T et al. *JAMA Network Open.*

eMethods

1. Sample description and exposure

We used data from the Massachusetts All-Payer Claims Database (Version 8.0) for January 2014 through December 31, 2018. These data included information on health insurance enrollment, medical claims, insurance product information, and clinician data for MassHealth (the combined Medicaid and state Children's Health Insurance Program in Massachusetts) and commercial payers including employer-sponsored insurance, some self-insured employers, health insurance marketplaces, and individually purchased plans.

We aggregated these data to the clinician-month level. eFigure 1 shows how we constructed our sample. First, we identified 3,759 clinicians as unique national providers identifiers (NPIs) whose primary practice location was in Massachusetts and who billed five or more well-child visits for children aged 1-5 years in 2014. We identified well-child visits using Current Procedural Terminology (CPT) codes 99381-3 and 99391-3 for new and established well-child medical visits to those to identify a population likely to see patients eligible for fluoride varnish based on recommendations. We excluded those who had missing or invalid values for other covariates (N=61). Finally, we required that clinicians were observed in the dataset both pre- and post-mandate, excluding clinicians entering dataset after the mandate (N=1,293). The final analytical sample included 107,841 clinician-month observations for 2,405 unique clinicians.

We defined clinician-months occurring on or after January 1, 2015 as exposed to the ACA mandate that private insurers cover fluoride varnish applications without cost-sharing, and considered those before January 1, 2015 as unexposed. We defined 2014 as our pre-mandate period because although the mandate began May 1, 2015, insurers typically operate on the calendar year.

2. Outcome variables

Because fluoride varnish is recommended to be applied during well-child medical visits, our primary outcome of interest was an indicator for whether a clinician applied fluoride varnish during at least one new or established well-child visit in a month (CPT codes 99381-3 and 99391-3). We identified fluoride varnish applications using CPT code 99188 and Current Dental Terminology (CDT) code D1206 and having the same service date as the well-child visit. Our second outcome is a monthly share of well-child visits that included fluoride varnish separately for clinicians who did and did not apply fluoride varnish pre-mandate in 2014.

3. Empirical approach

For each outcome, we estimated the following interrupted time-series regression model:

$$Y_{im} = \alpha + \sum_{-8}^{-1} \beta_{pre} + \sum_{1}^{36} \beta_{post} + \gamma_i + \theta' X_{im} + \epsilon_{im} \quad (1)$$

where *i* indexes the clinician, and *m* the calendar month. Y_{im} describes the outcome of interest, measured at the clinician-month level. The subscripts *pre* and *post* refer to monthly intervals respectively before and after the ACA mandate. For example, *pre* -6 refers to 6 months prior to December 2014. We combined month-periods 8 or more prior to the mandate and month-

© 2023 Gracner T et al. *JAMA Network Open.*

periods 36 or more after the mandate into a single bin, respectively. γ_i describes clinician-level fixed effects, controlling for their time-invariant unobservable characteristics, and X_{im} is a vector of additional controls (i.e., county-level measures of dentists per 1,000 population and pediatricians and family medicine physicians per 1,000 population <18 years old; a ZIP-code level measure of percentage of the population below 200% of the federal poverty level). β_{pre} describes relative differences in outcomes for months prior to the reference period (December 2014). β_{post} describes relative differences in outcomes for months after the reference period. All of these coefficients are plotted in our main and supplement figures with 95% confidence intervals, adjusted for serial correlation by clustering at the clinician level. We estimate this regression model overall and separately by clinician types defined by the type of insurance paying for visits. Four our secondary outcome which was the share of well-child visits with fluoride varnish application, we estimate these models separately for clinicians who did and did not apply fluoride varnish pre-mandate in 2014.

© 2023 Gracner T et al. *JAMA Network Open.*

eFigure 1: Analytic sample construction

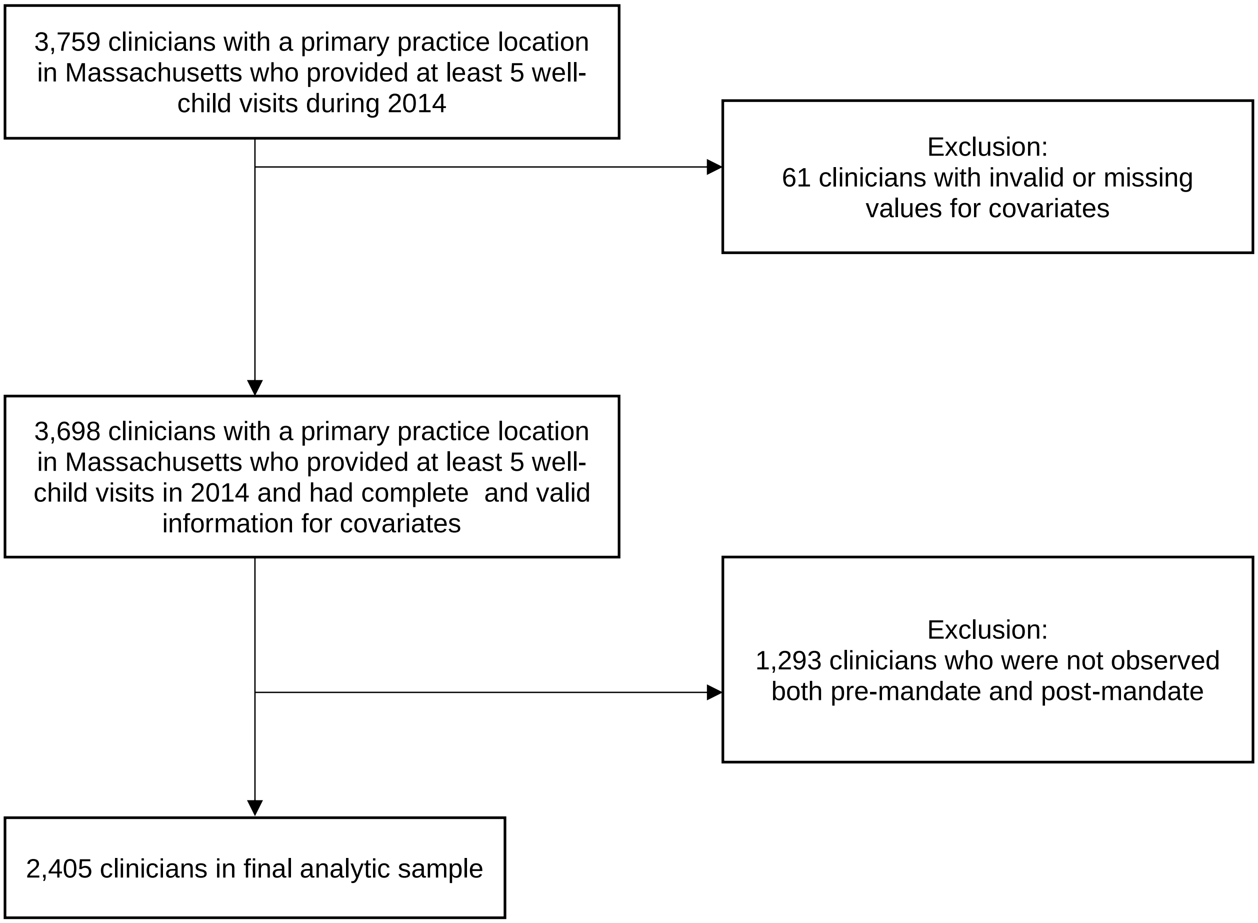

© 2023 Gracner T et al. *JAMA Network Open.*

eFigure 2: Unadjusted changes in fluoride varnish provision at well-child visits before and after December 2014 for clinicians providing no fluoride varnish pre-mandate

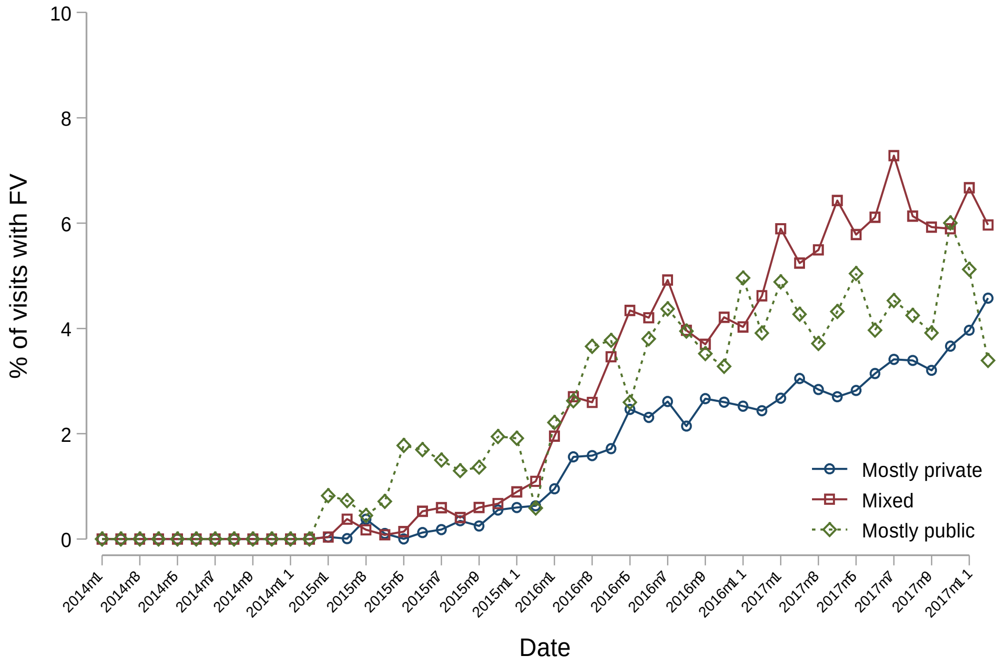

Notes: Clinicians were categorized based on tercile of well-child visits paid by private insurers: mostly private (>66% of visits paid by private insurers, blue line), mostly public (<33% of visits paid by private insurers, green line), and mixed (33-66% of visits paid by private insurers, red line). We defined months prior to January 2015 (gray solid vertical line) as our pre-mandate period because although the mandate began May 1, 2015 (gray dashed vertical line), insurers typically operate on the calendar year. FV, fluoride varnish.

© 2023 Gracner T et al. *JAMA Network Open.*

eFigure 3: Adjusted changes in FV applications during visits before and after April 2015

A: Change in any FV application during a visit per month

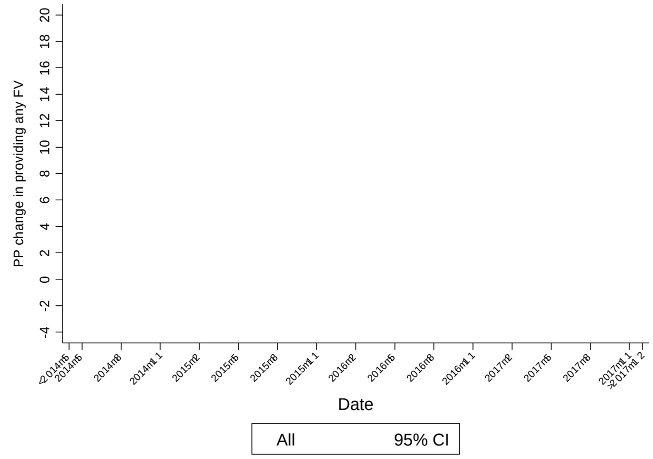

B: Change in share of visits with FV per month among clinicians who applied FV pre-mandate

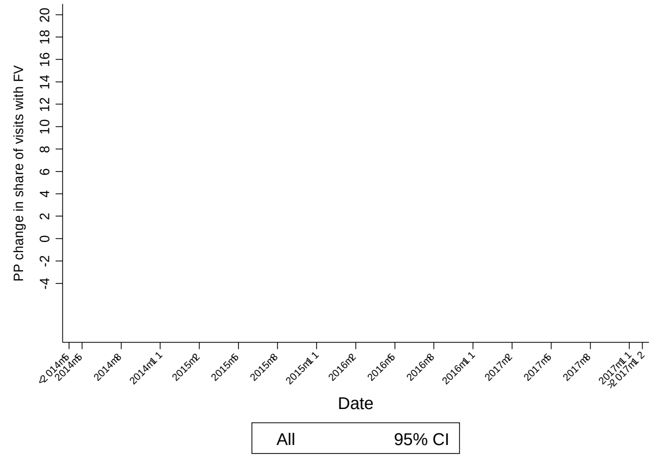

C: Change in share of visits with FV per month among clinicians who did not apply FV pre-mandate

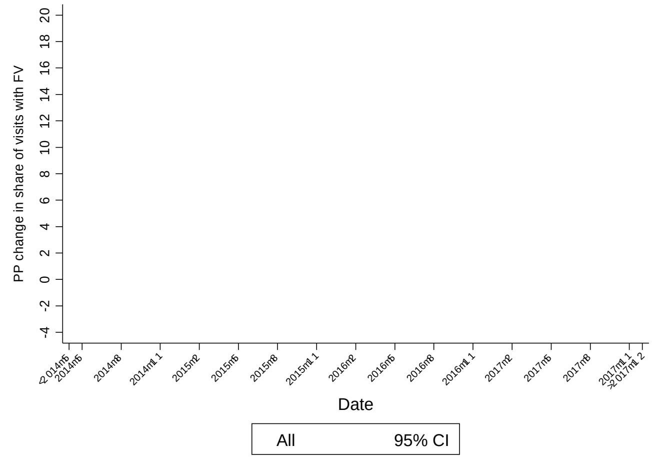

Notes: Markers represent the estimated associations with corresponding 95% CIs at each month relative to April 2015 (solid gray line indicates May 2015). Light gray dashed line indicates January 2015.

© 2023 Gracner T et al. *JAMA Network Open.*

eFigure 4: Adjusted changes in any fluoride varnish application at well-child visits relative to December 2014 using alternative specifications

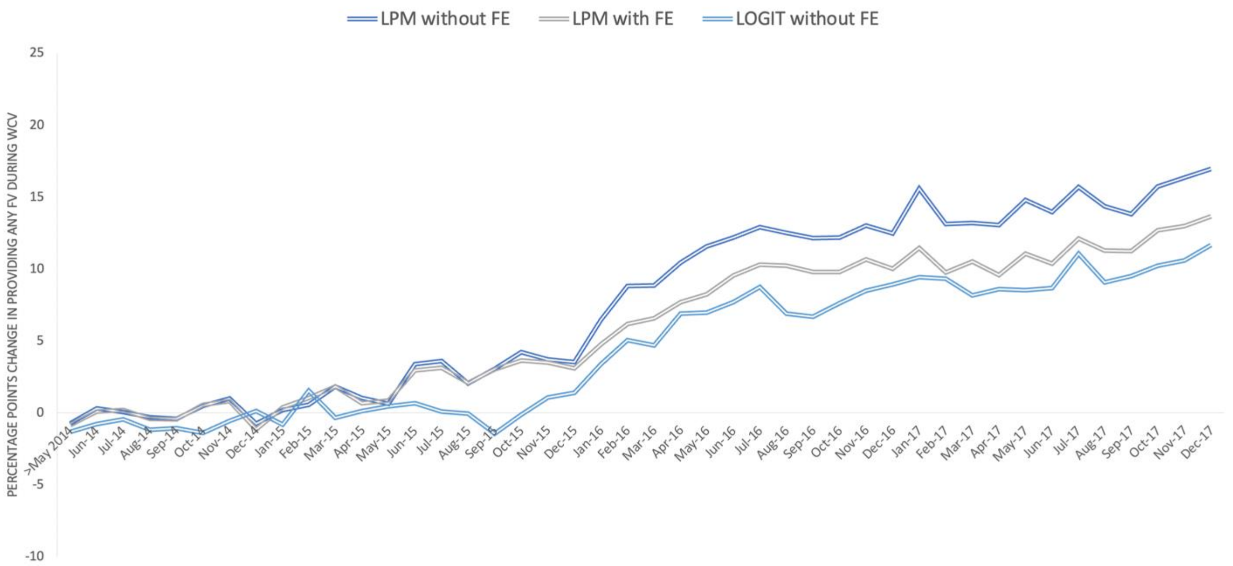

Notes: Results are presented for regression models estimating adjusted monthly changes in any fluoride varnish application at well-child visits relative to December 2014 using alternative specifications. We present coefficient estimates from a linear probability model (LPM) with clinician fixed effects (the preferred model presented throughout the manuscript) and a LPM without clinician fixed effects. We also present adjusted marginal effects from a logit model estimated LPM without clinician fixed effects. This figure illustrates that the adjusted monthly changes in any fluoride varnish application relative to December 2014 are similar across models.

© 2023 Gracner T et al. *JAMA Network Open.*

eFigure 5. Adjusted changes in FV applications during visits before and after December 2014, sensitivity analysis allowing clinicians to enter the dataset post-mandate

A: Change in any FV application during a visit per month

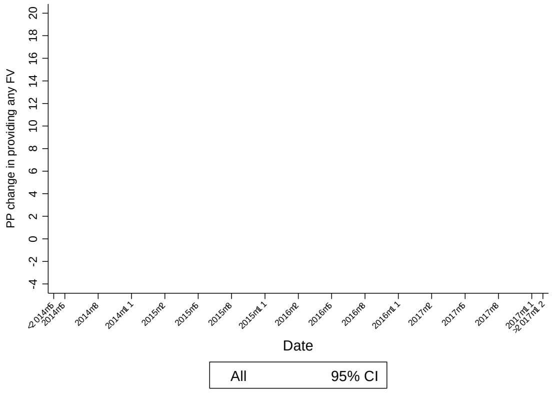

B: Change in share of visits with FV per month among clinicians who applied FV pre-mandate

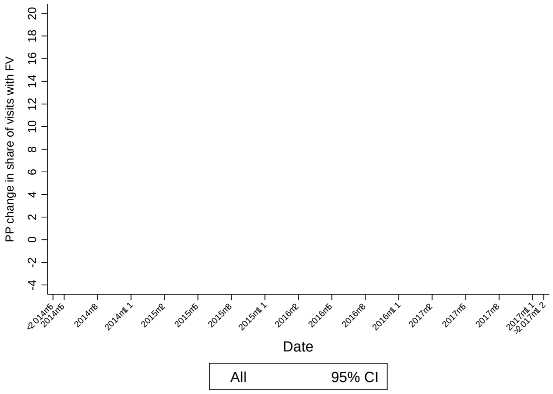

C: Change in share of visits with FV per month among clinicians who did not apply FV pre-mandate

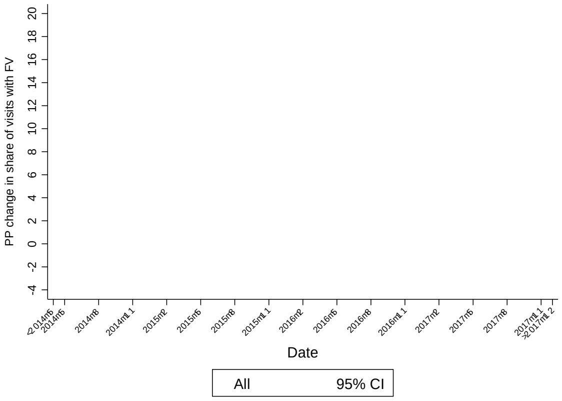

Notes: Markers represent the estimated associations with corresponding 95% CIs at each month relative to April 2015 (solid gray line indicates May 2015). Light gray dashed line indicates January 2015.

© 2023 Gracner T et al. *JAMA Network Open.*

eFigure 6: Adjusted changes in FV applications during visits before and after December 2014, sensitivity analysis that removed the restriction to include only clinicians with at least 5 well-child visits pre-mandate

A: Change in any FV application during a visit per month

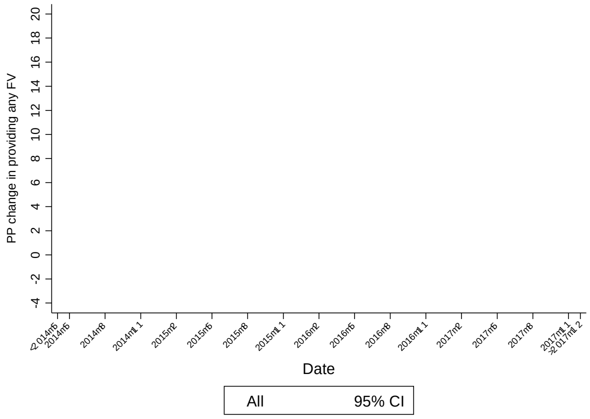

B: Change in share of visits with FV per month among clinicians who applied FV pre-mandate

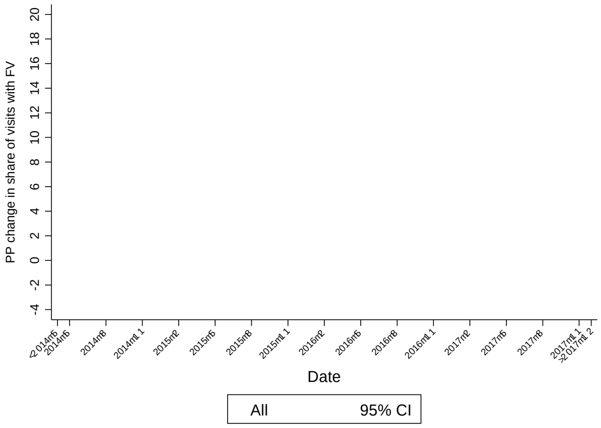

C: Change in share of visits with FV per month among clinicians who did not apply FV pre-mandate

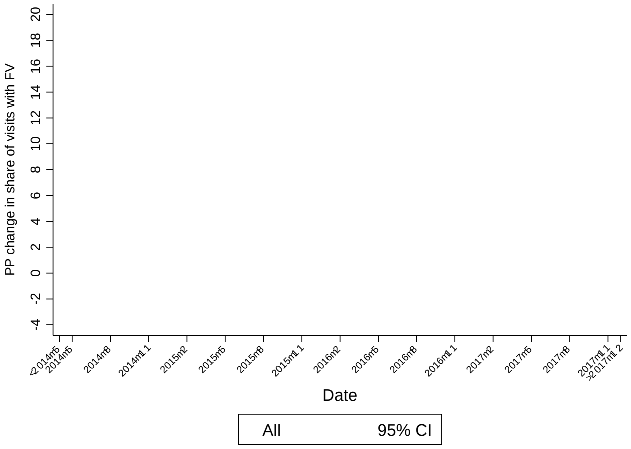

Notes: Markers represent the estimated associations with corresponding 95% CIs at each month relative to April 2015 (solid gray line indicates May 2015). Light gray dashed line indicates January 2015.

© 2023 Gracner T et al. *JAMA Network Open.*

eTable 1: Taxonomies of clinicians in study sample

	All Clinicians (%)	Clinicians serving mostly publicly insured patients	Clinicians serving both publicly and privately insured patients	Clinicians serving mostly privately insured patients
Total sample	2,405 (100%)	1,086 (100%)	655 (100%)	664 (100%)
Pediatricians	1,331 (55%)	709 (65%)	360 (55%)	243 (37%)
Family practice physicians	757 (31%)	252 (23%)	214 (33%)	278 (42%)
Other physicians	106 (4%)	28 (3%)	31 (5%)	41 (6%)
Other clinicians (e.g., NPs and PAs)	249 (10%)	97 (9%)	50 (7%)	102 (15%)

Note. Column percentages may not add to 100% due to rounding. Taxonomies of clinicians were identified based on the primary taxonomy reported in the National Plan and Provider Enumeration System.

© 2023 Gracner T et al. *JAMA Network Open.*

eTable 2: Regression model results that correspond to Figure 2.

**Exhibits within Figures 2:**		***2A***	***2B***	***2C***
**Outcome:**		***FV at least once during WCV*** [b]	***%FV of all WCV (if FV>0 in 2014)*** [b]	***%FV of all WCV (if FV=0 in 2014)*** [b]
**1/2014-4/2014**	*Estimate*[a]	-0.842	0.00629	-0.111
	SE	(0.771)	(1.062)	(0.105)
	95%CI	-2.355 - 0.671	-2.082 - 2.095	-0.317 - 0.0944
	p-value	(0.275)	(0.995)	(0.289)
**May-14**	*Estimate*	0.108	0.256	-0.117
	SE	(0.828)	(1.105)	(0.115)
	95%CI	-1.515 - 1.731	-1.916 - 2.428	-0.343 - 0.108
	p-value	(0.896)	(0.817)	(0.308)
**Jun-14**	*Estimate*	0.188	-0.468	-0.0219
	SE	(0.786)	(1.144)	(0.130)
	95%CI	-1.353 - 1.728	-2.717 - 1.781	-0.277 - 0.233
	p-value	(0.811)	(0.683)	(0.866)
**Jul-14**	*Estimate*	-0.427	-1.312	-0.0669
	SE	(0.775)	(1.093)	(0.113)
	95%CI	-1.947 - 1.094	-3.460 - 0.836	-0.288 - 0.155
	p-value	(0.582)	(0.231)	(0.554)
**Aug-14**	*Estimate*	-0.462	-1.661	-0.190
	SE	(0.846)	(1.085)	(0.128)
	95%CI	-2.121 - 1.198	-3.793 - 0.472	-0.442 - 0.0617
	p-value	(0.585)	(0.127)	(0.139)
**Sep-14**	*Estimate*	0.570	0.435	-0.117
	SE	(0.714)	(1.064)	(0.122)
	95%CI	-0.831 - 1.971	-1.656 - 2.527	-0.356 - 0.121
	p-value	(0.425)	(0.683)	(0.335)
**Oct-14**	*Estimate*	0.823	-0.757	-0.0623
	SE	(0.793)	(1.084)	(0.0911)
	95%CI	-0.733 - 2.379	-2.887 - 1.372	-0.241 - 0.116
	p-value	(0.300)	(0.485)	(0.494)
**Nov-14**	*Estimate*	-1.139	-0.535	-0.0263
	SE	(0.717)	(1.046)	(0.0952)
	95%CI	-2.546 - 0.268	-2.590 - 1.521	-0.213 - 0.160
	p-value	(0.112)	(0.609)	(0.782)
**Dec-14**	*Estimate*	0.395	2.387***	0.129
	SE	(0.724)	(0.919)	(0.184)
	95%CI	-1.024 - 1.814	0.579 - 4.194	-0.232 - 0.490
	p-value	(0.585)	(0.00977)	(0.484)
**Jan-15**	*Estimate*	1.020	4.326***	0.142
	SE	(0.662)	(1.046)	(0.138)
	95%CI	-0.278 - 2.318	2.270 - 6.383	-0.129 - 0.413
	p-value	(0.123)	(4.28e-05)	(0.305)
**Feb-15**	*Estimate*	1.843*	5.003***	0.0821
	SE	(0.723)	(1.132)	(0.183)
	95%CI	0.425 - 3.261	2.779 - 7.227	-0.276 - 0.440
	p-value	(0.0109)	(1.25e-05)	(0.653)
**Mar-15**	*Estimate*	0.661	3.748***	0.130
	SE	(0.753)	(1.186)	(0.155)
	95%CI	-0.816 - 2.138	1.418 - 6.079	-0.173 - 0.433
	p-value	(0.380)	(0.00168)	(0.400)
**May-15**	*Estimate*	0.850	3.322***	0.131
	SE	(0.674)	(1.142)	(0.121)
	95%CI	-0.472 - 2.171	1.078 - 5.566	-0.105 - 0.368
	p-value	(0.207)	(0.00380)	(0.276)
**Jun-15**	*Estimate*	2.958***	4.017***	0.228
	SE	(0.777)	(1.296)	(0.122)
	95%CI	1.434 - 4.482	1.469 - 6.564	-0.0117 - 0.467
	p-value	(0.000144)	(0.00207)	(0.0623)

© 2023 Gracner T et al. *JAMA Network Open.*

Cont… Exhibits within Figures 2:		*2A*	*2B*	*2C*
**Outcome:**		***FV at least once during WCV*** [b]	***%FV of all WCV (if FV>0 in 2014)*** [b]	***%FV of all WCV (if FV=0 in 2014)*** [b]
**Jul-15**	*Estimate*	3.141***	5.742***	0.252*
	SE	(0.781)	(1.461)	(0.117)
	95%CI	1.610 - 4.672	2.870 - 8.614	0.0236 - 0.481
	p-value	(5.92e-05)	(9.91e-05)	(0.0306)
**Aug-15**	*Estimate*	2.094**	3.473***	0.299*
	SE	(0.791)	(1.342)	(0.149)
	95%CI	0.543 - 3.645	0.835 - 6.111	0.00715 - 0.590
	p-value	(0.00815)	(0.00999)	(0.0446)
**Sep-15**	*Estimate*	3.038***	3.841***	0.266
	SE	(0.803)	(1.405)	(0.149)
	95%CI	1.464 - 4.612	1.079 - 6.604	-0.0269 - 0.558
	p-value	(0.000157)	(0.00653)	(0.0751)
**Oct-15**	*Estimate*	3.639***	3.846**	0.405**
	SE	(0.863)	(1.490)	(0.154)
	95%CI	1.946 - 5.332	0.918 - 6.773	0.103 - 0.708
	p-value	(2.59e-05)	(0.0102)	(0.00873)
**Nov-15**	*Estimate*	3.514***	4.393***	0.588***
	SE	(0.808)	(1.460)	(0.165)
	95%CI	1.930 - 5.099	1.523 - 7.264	0.263 - 0.912
	p-value	(1.42e-05)	(0.00278)	(0.000387)
**Dec-15**	*Estimate*	3.111***	6.055***	0.638***
	SE	(0.890)	(1.527)	(0.192)
	95%CI	1.366 - 4.856	3.054 - 9.056	0.261 - 1.015
	p-value	(0.000481)	(8.58e-05)	(0.000925)
**Jan-16**	*Estimate*	4.767***	7.089***	1.541***
	SE	(0.913)	(1.674)	(0.359)
	95%CI	2.976 - 6.557	3.799 - 10.38	0.838 - 2.244
	p-value	(1.95e-07)	(2.80e-05)	(1.81e-05)
**Feb-16**	*Estimate*	6.193***	6.458***	2.030***
	SE	(1.049)	(1.626)	(0.358)
	95%CI	4.136 - 8.251	3.262 - 9.653	1.327 - 2.732
	p-value	(4.06e-09)	(8.38e-05)	(1.68e-08)
**Mar-16**	*Estimate*	6.569***	7.072***	2.095***
	SE	(1.027)	(1.547)	(0.385)
	95%CI	4.555 - 8.584	4.031 - 10.11	1.340 - 2.850
	p-value	(1.92e-10)	(6.40e-06)	(5.91e-08)
**Apr-16**	*Estimate*	7.689***	8.911***	2.365***
	SE	(1.045)	(1.718)	(0.361)
	95%CI	5.640 - 9.739	5.533 - 12.29	1.657 - 3.072
	p-value	(0)	(3.34e-07)	(7.09e-11)
**May-16**	*Estimate*	8.233***	10.17***	3.041***
	SE	(1.147)	(1.657)	(0.434)
	95%CI	5.984 - 10.48	6.912 - 13.43	2.191 - 3.892
	p-value	(0)	(1.94e-09)	(0)
**Jun-16**	*Estimate*	9.535***	9.061***	3.077***
	SE	(1.137)	(1.746)	(0.399)
	95%CI	7.306 - 11.76	5.630 - 12.49	2.295 - 3.859
	p-value	(0)	(3.26e-07)	(0)
**Jul-16**	*Estimate*	10.30***	9.741***	3.494***
	SE	(1.164)	(1.605)	(0.433)
	95%CI	8.021 - 12.58	6.586 - 12.90	2.644 - 4.344
	p-value	(0)	(2.88e-09)	(0)
**Aug-16**	*Estimate*	10.22***	7.858***	3.355***
	SE	(1.143)	(1.554)	(0.426)
	95%CI	7.976 - 12.46	4.803 - 10.91	2.519 - 4.190
	p-value	(0)	(6.40e-07)	(0)
**Sep-16**	*Estimate*	9.793***	8.684***	3.289***

© 2023 Gracner T et al. *JAMA Network Open.*

Cont… Exhibits within Figures 2:		2A	2B	2C
**Outcome:**		***FV at least once during WCV*** [b]	***%FV of all WCV (if FV>0 in 2014)*** [b]	***%FV of all WCV (if FV=0 in 2014)*** [b]
	SE	(1.143)	(1.607)	(0.402)
	95%CI	7.552 - 12.03	5.526 - 11.84	2.501 - 4.078
	p-value	(0)	(1.09e-07)	(0)
**Oct-16**	*Estimate*	9.805***	9.007***	3.569***
	SE	(1.116)	(1.590)	(0.440)
	95%CI	7.617 - 11.99	5.882 - 12.13	2.706 - 4.433
	p-value	(0)	(2.71e-08)	(0)
**Nov-16**	*Estimate*	10.67***	8.359***	3.731***
	SE	(1.142)	(1.696)	(0.458)
	95%CI	8.425 - 12.91	5.025 - 11.69	2.832 - 4.630
	p-value	(0)	(1.19e-06)	(0)
**Dec-16**	*Estimate*	10.01***	9.239***	3.601***
	SE	(1.174)	(1.760)	(0.484)
	95%CI	7.709 - 12.31	5.780 - 12.70	2.652 - 4.550
	p-value	(0)	(2.40e-07)	(0)
**Jan-17**	*Estimate*	11.48***	11.12***	4.229***
	SE	(1.308)	(1.786)	(0.506)
	95%CI	8.909 - 14.04	7.610 - 14.63	3.237 - 5.221
	p-value	(0)	(1.15e-09)	(0)
**Feb-17**	*Estimate*	9.755***	9.394***	4.126***
	SE	(1.280)	(1.834)	(0.494)
	95%CI	7.246 - 12.26	5.788 - 13.00	3.157 - 5.096
	p-value	(0)	(4.62e-07)	(0)
**Mar-17**	*Estimate*	10.52***	9.353***	4.244***
	SE	(1.262)	(1.833)	(0.500)
	95%CI	8.043 - 12.99	5.751 - 12.96	3.262 - 5.225
	p-value	(0)	(5.04e-07)	(0)
**Apr-17**	*Estimate*	9.579***	9.486***	4.366***
	SE	(1.271)	(1.894)	(0.508)
	95%CI	7.086 - 12.07	5.762 - 13.21	3.369 - 5.363
	p-value	(0)	(8.10e-07)	(0)
**May-17**	*Estimate*	11.07***	8.816***	4.208***
	SE	(1.271)	(1.856)	(0.490)
	95%CI	8.583 - 13.57	5.167 - 12.46	3.246 - 5.170
	p-value	(0)	(2.80e-06)	(0)
**Jun-17**	*Estimate*	10.39***	9.184***	4.465***
	SE	(1.260)	(1.840)	(0.478)
	95%CI	7.919 - 12.86	5.568 - 12.80	3.527 - 5.403
	p-value	(0)	(8.74e-07)	(0)
**Jul-17**	*Estimate*	12.13***	9.968***	4.858***
	SE	(1.289)	(1.709)	(0.527)
	95%CI	9.600 - 14.65	6.608 - 13.33	3.825 - 5.891
	p-value	(0)	(1.09e-08)	(0)
**Aug-17**	*Estimate*	11.28***	7.214***	4.689***
	SE	(1.277)	(1.736)	(0.518)
	95%CI	8.776 - 13.79	3.801 - 10.63	3.674 - 5.704
	p-value	(0)	(3.95e-05)	(0)
**Sep-17**	*Estimate*	11.24***	8.034***	4.490***
	SE	(1.321)	(1.711)	(0.487)
	95%CI	8.648 - 13.83	4.671 - 11.40	3.534 - 5.445
	p-value	(0)	(3.60e-06)	(0)
**Oct-17**	*Estimate*	12.71***	8.505***	4.601***
	SE	(1.371)	(1.860)	(0.469)
	95%CI	10.02 - 15.40	4.848 - 12.16	3.681 - 5.520
	p-value	(0)	(6.35e-06)	(0)
**Nov-17**	*Estimate*	12.96***	7.143***	5.415***
	SE	(1.416)	(1.750)	(0.529)

© 2023 Gracner T et al. *JAMA Network Open.*

	95%CI	10.19 - 15.74	3.704 - 10.58	4.378 - 6.452
**Cont…Exhibits within Figures 2:**		***2A***	***2B***	***2C***
**Outcome:**		***FV at least once during WCV*** [b]	***%FV of all WCV (if FV>0 in 2014)*** [b]	***%FV of all WCV (if FV=0 in 2014)*** [b]
	p-value	(0)	(5.32e-05)	(0)
**Dec-17**	*Estimate*	13.64***	9.215***	5.660***
	SE	(1.364)	(1.936)	(0.577)
	95%CI	10.97 - 16.32	5.410 - 13.02	4.528 - 6.792
	p-value	(0)	(2.66e-06)	(0)
**Year 2018**	*Estimate*	21.50***	8.949***	8.802***
	SE	(1.394)	(1.622)	(0.585)
	95%CI	18.76 - 24.23	5.761 - 12.14	7.654 - 9.951
	p-value	(0)	(6.01e-08)	(0)
No. observations		107,841	19,583	88,258

Notes:

[a)] Standard errors are robust and clustered at the clinician level. */**/*** implies significantly different at p<0.05/0.01/0.001.

[b)] Controls included were physician fixed effects, county-level measures of dentists per 1,000 population and pediatricians and family medicine physicians per 1,000 population <18 years old; a ZIP-code level measure of percentage of the population below 200% of the federal poverty level.

© 2023 Gracner T et al. *JAMA Network Open.*

eTable 3: Regression model results that correspond to Figure 3A.

		Mostly Public	Mixed	Mostly Private
**Outcome:**		***FV at least once during WCV*** [b]	***FV at least once during WCV*** [b]	***FV at least once during WCV*** [b]
**Model:**		LPM	LPM	LPM
**1/2014-4/2014**	*Estimate* [a]	-0.152	-0.125	-1.522*
	SE	(2.105)	(1.987)	(0.664)
	95%CI	-4.285 - 3.981	-4.027 - 3.777	-2.826 - -0.218
	p-value	(0.942)	(0.950)	(0.0222)
**May-14**	*Estimate*	1.265	0.0994	-0.376
	SE	(2.298)	(2.120)	(0.735)
	95%CI	-3.246 - 5.777	-4.063 - 4.262	-1.818 - 1.066
	p-value	(0.582)	(0.963)	(0.609)
**Jun-14**	*Estimate*	-0.286	0.244	0.293
	SE	(2.379)	(1.985)	(0.637)
	95%CI	-4.956 - 4.385	-3.653 - 4.141	-0.957 - 1.542
	p-value	(0.904)	(0.902)	(0.646)
**Jul-14**	*Estimate*	-3.015	-1.050	0.833
	SE	(2.112)	(1.968)	(0.701)
	95%CI	-7.162 - 1.133	-4.914 - 2.813	-0.542 - 2.209
	p-value	(0.154)	(0.594)	(0.235)
**Aug-14**	*Estimate*	-4.734*	0.388	0.678
	SE	(2.341)	(1.879)	(0.893)
	95%CI	-9.331 - -0.138	-3.301 - 4.078	-1.074 - 2.430
	p-value	(0.0435)	(0.836)	(0.448)
**Sep-14**	*Estimate*	-1.533	2.248	0.482
	SE	(2.167)	(1.634)	(0.683)
	95%CI	-5.787 - 2.721	-0.961 - 5.457	-0.857 - 1.821
	p-value	(0.480)	(0.170)	(0.480)
**Oct-14**	*Estimate*	-0.601	2.168	0.604
	SE	(2.655)	(1.651)	(0.740)
	95%CI	-5.814 - 4.612	-1.074 - 5.410	-0.848 - 2.055
	p-value	(0.821)	(0.190)	(0.415)
**Nov-14**	*Estimate*	-2.926	-1.743	-0.228
	SE	(2.142)	(1.744)	(0.624)
	95%CI	-7.132 - 1.279	-5.167 - 1.682	-1.451 - 0.995
	p-value	(0.172)	(0.318)	(0.715)
**Dec-14**	*Estimate*	-1.456	1.161	0.640
	SE	(2.101)	(1.606)	(0.781)
	95%CI	-5.582 - 2.671	-1.992 - 4.314	-0.893 - 2.173
	p-value	(0.489)	(0.470)	(0.413)
**Jan-15**	*Estimate*	2.854	-0.345	1.144
	SE	(2.049)	(1.496)	(0.637)
	95%CI	-1.168 - 6.877	-3.282 - 2.592	-0.105 - 2.394
	p-value	(0.164)	(0.818)	(0.0726)
**Feb-15**	*Estimate*	3.435	2.167	1.082
	SE	(2.318)	(1.525)	(0.697)
	95%CI	-1.115 - 7.986	-0.826 - 5.161	-0.285 - 2.448
	p-value	(0.139)	(0.156)	(0.121)
**Mar-15**	*Estimate*	2.720	-0.430	0.468
	SE	(2.572)	(1.436)	(0.735)
	95%CI	-2.331 - 7.770	-3.250 - 2.389	-0.975 - 1.910
	p-value	(0.291)	(0.764)	(0.525)
**May-15**	*Estimate*	1.652	-0.162	1.215
	SE	(1.889)	(1.422)	(0.776)
	95%CI	-2.056 - 5.361	-2.954 - 2.629	-0.308 - 2.738
	p-value	(0.382)	(0.909)	(0.118)
**Jun-15**	*Estimate*	3.787	2.974	2.703**
	SE	(2.200)	(1.591)	(0.894)
	95%CI	-0.532 - 8.106	-0.150 - 6.099	0.948 - 4.458
	p-value	(0.0856)	(0.0620)	(0.00257)
**Jul-15**	*Estimate*	3.811	3.505*	2.828**

© 2023 Gracner T et al. *JAMA Network Open.*

Cont…		Mostly Public	Mixed	Mostly Private
**Outcome:**		***FV at least once during WCV*** [b)]	***FV at least once during WCV*** [b)]	***FV at least once during WCV*** [b)]
**Model:**		LPM	LPM	LPM
	SE	(2.300)	(1.649)	(0.865)
	95%CI	-0.705 - 8.326	0.266 - 6.743	1.130 - 4.526
	p-value	(0.0980)	(0.0340)	(0.00111)
**Aug-15**	*Estimate*	4.086	1.415	1.878*
	SE	(2.439)	(1.773)	(0.774)
	95%CI	-0.703 - 8.875	-2.065 - 4.896	0.360 - 3.396
	p-value	(0.0943)	(0.425)	(0.0153)
**Sep-15**	*Estimate*	3.563	4.811**	2.153**
	SE	(2.635)	(1.643)	(0.830)
	95%CI	-1.611 - 8.738	1.586 - 8.036	0.524 - 3.782
	p-value	(0.177)	(0.00352)	(0.00965)
**Oct-15**	*Estimate*	5.584*	3.862*	3.004***
	SE	(2.803)	(1.825)	(0.855)
	95%CI	0.0806 - 11.09	0.277 - 7.446	1.325 - 4.682
	p-value	(0.0467)	(0.0348)	(0.000463)
**Nov-15**	*Estimate*	1.779	4.514**	3.760***
	SE	(2.707)	(1.667)	(0.816)
	95%CI	-3.536 - 7.094	1.240 - 7.788	2.158 - 5.362
	p-value	(0.511)	(0.00696)	(4.61e-06)
**Dec-15**	*Estimate*	3.292	4.103*	2.710**
	SE	(2.709)	(1.937)	(0.927)
	95%CI	-2.028 - 8.612	0.299 - 7.906	0.891 - 4.529
	p-value	(0.225)	(0.0346)	(0.00354)
**Jan-16**	*Estimate*	4.903	5.403**	4.422***
	SE	(2.639)	(1.929)	(0.992)
	95%CI	-0.278 - 10.08	1.615 - 9.191	2.476 - 6.368
	p-value	(0.0636)	(0.00524)	(9.11e-06)
**Feb-16**	*Estimate*	6.588*	8.270***	4.756***
	SE	(3.097)	(2.177)	(1.070)
	95%CI	0.507 - 12.67	3.995 - 12.55	2.657 - 6.856
	p-value	(0.0338)	(0.000159)	(9.63e-06)
**Mar-16**	*Estimate*	5.897*	9.568***	4.866***
	SE	(2.948)	(2.221)	(0.992)
	95%CI	0.109 - 11.69	5.206 - 13.93	2.921 - 6.812
	p-value	(0.0459)	(1.90e-05)	(1.06e-06)
**Apr-16**	*Estimate*	5.309	12.34***	5.776***
	SE	(2.756)	(2.261)	(1.137)
	95%CI	-0.101 - 10.72	7.903 - 16.78	3.546 - 8.006
	p-value	(0.0544)	(6.81e-08)	(4.40e-07)
**May-16**	*Estimate*	3.673	13.55***	7.004***
	SE	(3.472)	(2.362)	(1.133)
	95%CI	-3.145 - 10.49	8.909 - 18.18	4.781 - 9.228
	p-value	(0.290)	(1.49e-08)	(9.01e-10)
**Jun-16**	*Estimate*	6.961*	13.78***	7.969***
	SE	(3.408)	(2.290)	(1.194)
	95%CI	0.268 - 13.65	9.282 - 18.27	5.626 - 10.31
	p-value	(0.0415)	(2.96e-09)	(0)
**Jul-16**	*Estimate*	8.431*	15.02***	8.112***
	SE	(3.300)	(2.446)	(1.245)
	95%CI	1.951 - 14.91	10.22 - 19.83	5.670 - 10.55
	p-value	(0.0108)	(1.41e-09)	(1.10e-10)
**Aug-16**	*Estimate*	8.850**	14.59***	7.927***
	SE	(3.242)	(2.328)	(1.254)
	95%CI	2.484 - 15.22	10.02 - 19.16	5.467 - 10.39
	p-value	(0.00651)	(6.69e-10)	(3.78e-10)
**Sep-16**	*Estimate*	5.907	14.36***	8.721***
	SE	(3.433)	(2.345)	(1.216)
	95%CI	-0.833 - 12.65	9.754 - 18.96	6.335 - 11.11

© 2023 Gracner T et al. *JAMA Network Open.*

Cont…		Mostly Public	Mixed	Mostly Private
**Outcome:**		***FV at least once during WCV*** b)	***FV at least once during WCV*** b)	***FV at least once during WCV*** b)
**Model:**		LPM	LPM	LPM
	p-value	(0.0858)	(1.58e-09)	(0)
**Oct-16**	*Estimate*	6.652*	14.67***	8.202***
	SE	(3.093)	(2.391)	(1.216)
	95%CI	0.578 - 12.73	9.978 - 19.37	5.816 - 10.59
	p-value	(0.0319)	(1.46e-09)	(0)
**Nov-16**	*Estimate*	5.066	16.54***	9.534***
	SE	(3.085)	(2.374)	(1.317)
	95%CI	-0.993 - 11.12	11.88 - 21.20	6.949 - 12.12
	p-value	(0.101)	(0)	(0)
**Dec-16**	*Estimate*	4.214	15.23***	9.330***
	SE	(3.237)	(2.475)	(1.278)
	95%CI	-2.143 - 10.57	10.37 - 20.09	6.822 - 11.84
	p-value	(0.193)	(1.31e-09)	(0)
**Jan-17**	*Estimate*	7.915*	16.78***	9.596***
	SE	(3.909)	(2.578)	(1.377)
	95%CI	0.240 - 15.59	11.72 - 21.84	6.895 - 12.30
	p-value	(0.0433)	(1.52e-10)	(0)
**Feb-17**	*Estimate*	3.644	16.48***	8.278***
	SE	(3.864)	(2.580)	(1.257)
	95%CI	-3.943 - 11.23	11.41 - 21.54	5.812 - 10.74
	p-value	(0.346)	(3.25e-10)	(6.95e-11)
**Mar-17**	*Estimate*	3.843	17.39***	9.159***
	SE	(3.715)	(2.528)	(1.323)
	95%CI	-3.452 - 11.14	12.42 - 22.35	6.563 - 11.75
	p-value	(0.301)	(0)	(0)
**Apr-17**	*Estimate*	0.454	18.55***	8.087***
	SE	(3.769)	(2.613)	(1.235)
	95%CI	-6.946 - 7.853	13.42 - 23.68	5.664 - 10.51
	p-value	(0.904)	(0)	(8.97e-11)
**May-17**	*Estimate*	3.806	17.99***	10.02***
	SE	(3.615)	(2.632)	(1.340)
	95%CI	-3.292 - 10.90	12.83 - 23.16	7.391 - 12.65
	p-value	(0.293)	(0)	(0)
**Jun-17**	*Estimate*	2.341	17.78***	9.540***
	SE	(3.444)	(2.593)	(1.402)
	95%CI	-4.421 - 9.104	12.69 - 22.87	6.789 - 12.29
	p-value	(0.497)	(0)	(0)
**Jul-17**	*Estimate*	4.170	19.35***	11.23***
	SE	(3.298)	(2.726)	(1.477)
	95%CI	-2.306 - 10.65	13.99 - 24.70	8.331 - 14.13
	p-value	(0.207)	(0)	(0)
**Aug-17**	*Estimate*	4.712	17.55***	10.32***
	SE	(3.344)	(2.708)	(1.457)
	95%CI	-1.854 - 11.28	12.23 - 22.87	7.463 - 13.18
	p-value	(0.159)	(1.81e-10)	(0)
**Sep-17**	*Estimate*	2.106	17.69***	11.24***
	SE	(3.955)	(2.644)	(1.406)
	95%CI	-5.660 - 9.873	12.49 - 22.88	8.486 - 14.00
	p-value	(0.595)	(0)	(0)
**Oct-17**	*Estimate*	2.994	18.57***	13.34***
	SE	(3.684)	(2.731)	(1.648)
	95%CI	-4.239 - 10.23	13.21 - 23.93	10.11 - 16.58
	p-value	(0.417)	(0)	(0)
**Nov-17**	*Estimate*	-0.516	20.20***	14.48***
	SE	(3.787)	(2.795)	(1.680)
	95%CI	-7.953 - 6.920	14.71 - 25.69	11.18 - 17.77
	p-value	(0.892)	(0)	(0)
**Dec-17**	*Estimate*	2.154	19.47***	15.19***

© 2023 Gracner T et al. *JAMA Network Open.*

Cont…		Mostly Public	Mixed	Mostly Private
**Outcome:**		***FV at least once during WCV*** [b)]	***FV at least once during WCV*** [b)]	***FV at least once during WCV*** [b)]
**Model:**		LPM	LPM	LPM
	SE	(3.396)	(2.773)	(1.692)
	95%CI	-4.515 - 8.824	14.02 - 24.91	11.87 - 18.51
	p-value	(0.526)	(0)	(0)
**Year 2018**	*Estimate*	8.752**	23.89***	25.23***
	SE	(3.383)	(2.864)	(1.732)
	95%CI	2.109 - 15.39	18.26 - 29.51	21.83 - 28.63
	p-value	(0.00990)	(0)	(0)
No. observations		25,782	30,140	51,919

Notes:

[a)] Standard errors are robust and clustered at the clinician level. */**/*** implies significantly different at p<0.05/0.01/0.001.

[b)] Controls included were physician fixed effects, county-level measures of dentists per 1,000 population and pediatricians and family medicine physicians per 1,000 population <18 years old; a ZIP-code level measure of percentage of the population below 200% of the federal poverty level.

© 2023 Gracner T et al. *JAMA Network Open.*

eTable 4: Regression model results that correspond to Figure 3B.

		Mostly Public	*Mixed*	*Mostly Private*
**Outcome:**		***%FV of all WCV (if FV>0 in 2014)*** [b]	***%FV of all WCV (if FV>0 in 2014)*** [b]	***%FV of all WCV (if FV>0 in 2014)*** [b]
**Model:**		FE	FE	FE
**1/2014-4/2014**	*Estimate* [a]	0.713	0.476	-2.793*
	SE	(1.890)	(1.433)	(1.211)
	95%CI	-3.008 - 4.433	-2.365 - 3.316	-5.218 - -0.367
	p-value	(0.706)	(0.741)	(0.0248)
**May-14**	*Estimate*	0.687	-0.170	-0.700
	SE	(1.908)	(1.556)	(1.539)
	95%CI	-3.070 - 4.444	-3.255 - 2.915	-3.782 - 2.382
	p-value	(0.719)	(0.913)	(0.651)
**Jun-14**	*Estimate*	-0.496	-0.283	-0.151
	SE	(2.110)	(1.341)	(1.555)
	95%CI	-4.650 - 3.658	-2.942 - 2.377	-3.265 - 2.963
	p-value	(0.814)	(0.833)	(0.923)
**Jul-14**	*Estimate*	-2.365	-0.391	0.406
	SE	(1.969)	(1.461)	(1.107)
	95%CI	-6.242 - 1.512	-3.289 - 2.506	-1.811 - 2.624
	p-value	(0.231)	(0.789)	(0.715)
**Aug-14**	*Estimate*	-3.526	-0.509	1.359
	SE	(1.962)	(1.269)	(1.508)
	95%CI	-7.388 - 0.337	-3.025 - 2.006	-1.661 - 4.379
	p-value	(0.0734)	(0.689)	(0.371)
**Sep-14**	*Estimate*	-0.464	1.414	0.985
	SE	(1.953)	(1.225)	(1.317)
	95%CI	-4.310 - 3.381	-1.015 - 3.843	-1.653 - 3.623
	p-value	(0.812)	(0.251)	(0.458)
**Oct-14**	*Estimate*	-1.198	-0.0781	-0.860
	SE	(2.065)	(1.115)	(1.159)
	95%CI	-5.265 - 2.869	-2.290 - 2.133	-3.182 - 1.461
	p-value	(0.562)	(0.944)	(0.461)
**Nov-14**	*Estimate*	-0.743	0.652	-1.739
	SE	(1.912)	(1.354)	(1.364)
	95%CI	-4.507 - 3.022	-2.032 - 3.337	-4.471 - 0.993
	p-value	(0.698)	(0.631)	(0.208)
**Dec-14**	*Estimate*	1.031	3.011**	3.977*
	SE	(1.565)	(1.109)	(1.979)
	95%CI	-2.049 - 4.112	0.812 - 5.209	0.0144 - 7.940
	p-value	(0.510)	(0.00775)	(0.0492)
**Jan-15**	*Estimate*	3.150*	4.215**	6.719*
	SE	(1.365)	(1.470)	(3.192)
	95%CI	0.462 - 5.839	1.300 - 7.129	0.328 - 13.11
	p-value	(0.0218)	(0.00500)	(0.0397)
**Feb-15**	*Estimate*	2.593	5.532***	9.313**
	SE	(1.542)	(1.460)	(3.226)
	95%CI	-0.443 - 5.629	2.636 - 8.427	2.853 - 15.77
	p-value	(0.0938)	(0.000252)	(0.00549)
**Mar-15**	*Estimate*	2.437	2.618	7.920*
	SE	(1.695)	(1.409)	(3.305)
	95%CI	-0.901 - 5.775	-0.176 - 5.412	1.303 - 14.54
	p-value	(0.152)	(0.0660)	(0.0199)
**May-15**	*Estimate*	1.456	2.605	8.779**
	SE	(1.663)	(1.589)	(2.869)
	95%CI	-1.819 - 4.732	-0.546 - 5.757	3.035 - 14.52
	p-value	(0.382)	(0.104)	(0.00337)
**Jun-15**	*Estimate*	-0.284	6.831***	9.382**
	SE	(2.020)	(1.645)	(2.956)
	95%CI	-4.262 - 3.693	3.570 - 10.09	3.463 - 15.30
	p-value	(0.888)	(6.67e-05)	(0.00242)
**Jul-15**	*Estimate*	0.639	9.928***	10.61***

© 2023 Gracner T et al. *JAMA Network Open.*

Cont…		Mostly Public	Mixed	Mostly Private
**Outcome:**		*%FV of all WCV (if FV>0 in 2014)* [b]	*%FV of all WCV (if FV>0 in 2014)* [b]	*%FV of all WCV (if FV>0 in 2014)* [b]
**Model:**		FE	FE	FE
	SE	(2.126)	(2.493)	(2.867)
	95%CI	-3.548 - 4.825	4.985 - 14.87	4.869 - 16.35
	p-value	(0.764)	(0.000125)	(0.000486)
**Aug-15**	*Estimate*	0.0439	6.720**	6.332**
	SE	(2.089)	(2.308)	(2.226)
	95%CI	-4.070 - 4.157	2.145 - 11.30	1.874 - 10.79
	p-value	(0.983)	(0.00438)	(0.00617)
**Sep-15**	*Estimate*	-0.600	6.948**	9.132**
	SE	(2.159)	(2.216)	(2.852)
	95%CI	-4.852 - 3.652	2.555 - 11.34	3.422 - 14.84
	p-value	(0.781)	(0.00222)	(0.00223)
**Oct-15**	*Estimate*	-2.294	8.190**	10.77**
	SE	(2.062)	(2.447)	(3.145)
	95%CI	-6.355 - 1.767	3.339 - 13.04	4.469 - 17.06
	p-value	(0.267)	(0.00113)	(0.00115)
**Nov-15**	*Estimate*	-1.579	8.986***	10.71***
	SE	(2.156)	(2.377)	(2.706)
	95%CI	-5.825 - 2.667	4.274 - 13.70	5.289 - 16.13
	p-value	(0.465)	(0.000259)	(0.000213)
**Dec-15**	*Estimate*	0.337	12.48***	9.201**
	SE	(2.311)	(2.459)	(2.977)
	95%CI	-4.213 - 4.887	7.601 - 17.35	3.239 - 15.16
	p-value	(0.884)	(1.68e-06)	(0.00309)
**Jan-16**	*Estimate*	0.0490	13.90***	12.27***
	SE	(2.442)	(3.075)	(2.926)
	95%CI	-4.759 - 4.857	7.803 - 20.00	6.415 - 18.13
	p-value	(0.984)	(1.62e-05)	(9.64e-05)
**Feb-16**	*Estimate*	-0.688	15.07***	9.233**
	SE	(2.270)	(3.005)	(2.687)
	95%CI	-5.159 - 3.782	9.115 - 21.03	3.853 - 14.61
	p-value	(0.762)	(2.14e-06)	(0.00111)
**Mar-16**	*Estimate*	-0.321	14.63***	12.34***
	SE	(2.206)	(2.606)	(3.067)
	95%CI	-4.665 - 4.024	9.467 - 19.80	6.198 - 18.48
	p-value	(0.885)	(1.59e-07)	(0.000171)
**Apr-16**	*Estimate*	0.547	17.58***	14.75***
	SE	(2.468)	(2.862)	(3.050)
	95%CI	-4.313 - 5.407	11.91 - 23.26	8.640 - 20.86
	p-value	(0.825)	(1.43e-08)	(1.05e-05)
**May-16**	*Estimate*	0.763	20.21***	16.36***
	SE	(2.423)	(2.720)	(2.890)
	95%CI	-4.007 - 5.534	14.81 - 25.60	10.57 - 22.15
	p-value	(0.753)	(0)	(5.12e-07)
**Jun-16**	*Estimate*	0.952	17.28***	14.73***
	SE	(2.499)	(2.942)	(3.252)
	95%CI	-3.969 - 5.874	11.44 - 23.11	8.219 - 21.24
	p-value	(0.704)	(5.00e-08)	(3.07e-05)
**Jul-16**	*Estimate*	1.547	18.45***	14.70***
	SE	(2.431)	(2.565)	(2.938)
	95%CI	-3.239 - 6.333	13.36 - 23.53	8.815 - 20.58
	p-value	(0.525)	(9.40e-11)	(5.74e-06)
**Aug-16**	*Estimate*	0.0106	15.59***	14.00***
	SE	(2.299)	(2.295)	(3.109)
	95%CI	-4.516 - 4.537	11.04 - 20.14	7.778 - 20.23
	p-value	(0.996)	(6.57e-10)	(3.35e-05)
**Sep-16**	*Estimate*	0.350	16.11***	16.31***
	SE	(2.531)	(2.294)	(3.015)
	95%CI	-4.634 - 5.334	11.56 - 20.66	10.27 - 22.35

© 2023 Gracner T et al. *JAMA Network Open.*

Cont…		Mostly Public	Mixed	Mostly Private
**Outcome:**		***%FV of all WCV (if FV>0 in 2014)*** [b]	***%FV of all WCV (if FV>0 in 2014)*** [b]	***%FV of all WCV (if FV>0 in 2014)*** [b]
**Model:**		FE	FE	FE
	p-value	(0.890)	(2.18e-10)	(1.30e-06)
**Oct-16**	*Estimate*	-0.880	20.08***	14.86***
	SE	(2.242)	(2.625)	(2.821)
	95%CI	-5.296 - 3.536	14.88 - 25.29	9.208 - 20.50
	p-value	(0.695)	(0)	(2.20e-06)
**Nov-16**	*Estimate*	-0.854	19.09***	13.09***
	SE	(2.336)	(3.087)	(2.785)
	95%CI	-5.455 - 3.747	12.97 - 25.21	7.517 - 18.67
	p-value	(0.715)	(1.20e-08)	(1.68e-05)
**Dec-16**	*Estimate*	-0.446	18.95***	16.52***
	SE	(2.456)	(2.905)	(3.516)
	95%CI	-5.283 - 4.391	13.19 - 24.71	9.482 - 23.57
	p-value	(0.856)	(2.39e-09)	(1.69e-05)
**Jan-17**	*Estimate*	1.111	22.15***	17.21***
	SE	(2.551)	(2.835)	(3.203)
	95%CI	-3.913 - 6.135	16.53 - 27.77	10.79 - 23.62
	p-value	(0.664)	(0)	(1.50e-06)
**Feb-17**	*Estimate*	-1.790	21.50***	17.61***
	SE	(2.465)	(2.843)	(3.375)
	95%CI	-6.644 - 3.063	15.87 - 27.14	10.86 - 24.37
	p-value	(0.468)	(0)	(2.62e-06)
**Mar-17**	*Estimate*	-1.688	21.60***	17.22***
	SE	(2.499)	(3.036)	(3.244)
	95%CI	-6.608 - 3.232	15.58 - 27.62	10.73 - 23.72
	p-value	(0.500)	(1.38e-10)	(1.88e-06)
**Apr-17**	*Estimate*	-1.828	20.53***	19.30***
	SE	(2.686)	(2.872)	(3.369)
	95%CI	-7.116 - 3.461	14.83 - 26.22	12.55 - 26.05
	p-value	(0.497)	(1.17e-10)	(3.99e-07)
**May-17**	*Estimate*	-1.758	19.51***	17.17***
	SE	(2.595)	(2.900)	(3.254)
	95%CI	-6.869 - 3.352	13.76 - 25.26	10.65 - 23.68
	p-value	(0.499)	(9.08e-10)	(2.14e-06)
**Jun-17**	*Estimate*	-0.0368	20.54***	13.37***
	SE	(2.605)	(2.923)	(3.275)
	95%CI	-5.167 - 5.094	14.75 - 26.34	6.812 - 19.93
	p-value	(0.989)	(2.12e-10)	(0.000140)
**Jul-17**	*Estimate*	2.294	18.65***	13.32***
	SE	(2.602)	(2.560)	(2.880)
	95%CI	-2.829 - 7.418	13.57 - 23.72	7.556 - 19.09
	p-value	(0.379)	(6.02e-11)	(2.19e-05)
**Aug-17**	*Estimate*	-1.178	16.23***	12.22***
	SE	(2.469)	(2.979)	(2.898)
	95%CI	-6.040 - 3.684	10.33 - 22.14	6.419 - 18.02
	p-value	(0.634)	(3.32e-07)	(8.94e-05)
**Sep-17**	*Estimate*	-2.067	19.61***	13.07***
	SE	(2.388)	(2.815)	(2.707)
	95%CI	-6.770 - 2.636	14.02 - 25.19	7.645 - 18.49
	p-value	(0.388)	(2.87e-10)	(1.08e-05)
**Oct-17**	*Estimate*	-1.420	19.03***	14.02***
	SE	(2.722)	(2.833)	(3.402)
	95%CI	-6.779 - 3.939	13.41 - 24.64	7.207 - 20.83
	p-value	(0.602)	(9.64e-10)	(0.000124)
**Nov-17**	*Estimate*	-3.403	16.75***	16.05***
	SE	(2.514)	(2.815)	(2.915)
	95%CI	-8.353 - 1.548	11.17 - 22.33	10.21 - 21.88
	p-value	(0.177)	(3.51e-08)	(9.16e-07)
**Dec-17**	*Estimate*	-1.557	20.78***	15.28***

© 2023 Gracner T et al. *JAMA Network Open.*

Cont…		Mostly Public	Mixed	Mostly Private
**Outcome:**		***%FV of all WCV (if FV>0 in 2014)*** [b]	***%FV of all WCV (if FV>0 in 2014)*** [b]	***%FV of all WCV (if FV>0 in 2014)*** [b]
**Model:**		FE	FE	FE
	SE	(2.840)	(3.141)	(2.920)
	95%CI	-7.151 - 4.036	14.55 - 27.01	9.429 - 21.12
	p-value	(0.584)	(1.55e-09)	(2.51e-06)
**Year 2018**	*Estimate*	0.0992	16.93***	15.76***
	SE	(2.030)	(3.072)	(2.754)
	95%CI	-3.897 - 4.096	10.84 - 23.02	10.24 - 21.27
	p-value	(0.961)	(2.52e-07)	(4.11e-07)
No. observations		10,980	5,392	3,211

Notes:

[a)] Standard errors are robust and clustered at the clinician level. */**/*** implies significantly different at p<0.05/0.01/0.001.

[b)] Controls included were physician fixed effects, county-level measures of dentists per 1,000 population and pediatricians and family medicine physicians per 1,000 population <18 years old; a ZIP-code level measure of percentage of the population below 200% of the federal poverty level.

© 2023 Gracner T et al. *JAMA Network Open.*

eTable 5: Regression model results that correspond to Figure 3C.

		Mostly Public	***Mixed***	***Mostly Private***
**Outcome:**		***%FV of all WCV (if FV=0 in 2014)***	***%FV of all WCV (if FV=0 in 2014)***	***%FV of all WCV (if FV=0 in 2014)***
**Model:**		FE	FE	FE
**1/2014-4/2014**	*Estimate*	-0.423	0.0126	-0.115
	SE	(0.280)	(0.261)	(0.111)
	95%CI	-0.974 - 0.128	-0.500 - 0.525	-0.332 - 0.102
	p-value	(0.132)	(0.962)	(0.298)
**May-14**	*Estimate*	-0.433	-0.0101	-0.136
	SE	(0.322)	(0.303)	(0.114)
	95%CI	-1.065 - 0.199	-0.606 - 0.585	-0.361 - 0.0877
	p-value	(0.179)	(0.973)	(0.233)
**Jun-14**	*Estimate*	-0.194	0.305	-0.161
	SE	(0.266)	(0.286)	(0.158)
	95%CI	-0.717 - 0.329	-0.257 - 0.867	-0.472 - 0.150
	p-value	(0.466)	(0.287)	(0.309)
**Jul-14**	*Estimate*	-0.305	0.0288	-0.0826
	SE	(0.265)	(0.294)	(0.120)
	95%CI	-0.825 - 0.216	-0.548 - 0.606	-0.317 - 0.152
	p-value	(0.251)	(0.922)	(0.490)
**Aug-14**	*Estimate*	-0.529	-0.0604	-0.179
	SE	(0.379)	(0.287)	(0.145)
	95%CI	-1.275 - 0.217	-0.625 - 0.504	-0.463 - 0.104
	p-value	(0.164)	(0.833)	(0.215)
**Sep-14**	*Estimate*	0.187	-0.146	-0.187
	SE	(0.267)	(0.234)	(0.157)
	95%CI	-0.338 - 0.711	-0.606 - 0.314	-0.495 - 0.121
	p-value	(0.484)	(0.533)	(0.235)
**Oct-14**	*Estimate*	0.0470	-0.121	-0.0749
	SE	(0.177)	(0.179)	(0.121)
	95%CI	-0.302 - 0.395	-0.472 - 0.231	-0.312 - 0.162
	p-value	(0.791)	(0.500)	(0.535)
**Nov-14**	*Estimate*	-0.117	0.0448	-0.0642
	SE	(0.216)	(0.214)	(0.112)
	95%CI	-0.541 - 0.306	-0.376 - 0.465	-0.284 - 0.156
	p-value	(0.586)	(0.834)	(0.567)
**Dec-14**	*Estimate*	1.123	-0.0284	-0.0251
	SE	(1.395)	(0.226)	(0.0977)
	95%CI	-1.618 - 3.865	-0.473 - 0.416	-0.217 - 0.167
	p-value	(0.421)	(0.900)	(0.797)
**Jan-15**	*Estimate*	0.838	0.0782	0.0299
	SE	(1.015)	(0.191)	(0.0867)
	95%CI	-1.156 - 2.833	-0.298 - 0.454	-0.140 - 0.200
	p-value	(0.409)	(0.683)	(0.731)
**Feb-15**	*Estimate*	1.454	-0.228	-0.0402
	SE	(1.337)	(0.232)	(0.0938)
	95%CI	-1.174 - 4.081	-0.683 - 0.228	-0.224 - 0.144
	p-value	(0.277)	(0.326)	(0.669)
**Mar-15**	*Estimate*	1.447	0.00596	-0.0652
	SE	(1.077)	(0.207)	(0.104)
	95%CI	-0.671 - 3.564	-0.400 - 0.412	-0.269 - 0.139
	p-value	(0.180)	(0.977)	(0.530)
**May-15**	*Estimate*	1.140	0.125	-0.0491
	SE	(0.620)	(0.189)	(0.134)
	95%CI	-0.0787 - 2.358	-0.247 - 0.496	-0.312 - 0.214
	p-value	(0.0667)	(0.511)	(0.714)
**Jun-15**	*Estimate*	1.533*	0.114	0.0556
	SE	(0.644)	(0.235)	(0.113)
	95%CI	0.267 - 2.799	-0.348 - 0.576	-0.166 - 0.277
	p-value	(0.0178)	(0.628)	(0.622)
**Jul-15**	*Estimate*	1.291**	0.434	0.0108

© 2023 Gracner T et al. *JAMA Network Open.*

Cont…		Mostly Public	Mixed	Mostly Private
**Outcome:**		*%FV of all WCV (if FV=0 in 2014)*	*%FV of all WCV (if FV=0 in 2014)*	*%FV of all WCV (if FV=0 in 2014)*
**Model:**		FE	FE	FE
	SE	(0.394)	(0.296)	(0.116)
	95%CI	0.515 - 2.066	-0.148 - 1.015	-0.217 - 0.238
	p-value	(0.00116)	(0.143)	(0.926)
**Aug-15**	*Estimate*	1.681	0.309	0.0637
	SE	(0.875)	(0.315)	(0.109)
	95%CI	-0.0387 - 3.401	-0.311 - 0.929	-0.150 - 0.278
	p-value	(0.0554)	(0.328)	(0.559)
**Sep-15**	*Estimate*	1.543	0.244	0.0742
	SE	(0.800)	(0.311)	(0.132)
	95%CI	-0.0297 - 3.115	-0.368 - 0.855	-0.184 - 0.333
	p-value	(0.0545)	(0.434)	(0.573)
**Oct-15**	*Estimate*	1.710*	0.306	0.231
	SE	(0.782)	(0.295)	(0.159)
	95%CI	0.173 - 3.247	-0.273 - 0.885	-0.0813 - 0.543
	p-value	(0.0293)	(0.299)	(0.147)
**Nov-15**	*Estimate*	2.057*	0.709*	0.309*
	SE	(0.948)	(0.338)	(0.139)
	95%CI	0.194 - 3.921	0.0443 - 1.373	0.0356 - 0.582
	p-value	(0.0306)	(0.0366)	(0.0268)
**Dec-15**	*Estimate*	1.849	0.559	0.492**
	SE	(1.127)	(0.348)	(0.179)
	95%CI	-0.367 - 4.066	-0.125 - 1.242	0.141 - 0.844
	p-value	(0.102)	(0.109)	(0.00606)
**Jan-16**	*Estimate*	3.802*	2.034**	0.907**
	SE	(1.871)	(0.666)	(0.319)
	95%CI	0.125 - 7.480	0.726 - 3.343	0.281 - 1.534
	p-value	(0.0428)	(0.00237)	(0.00458)
**Feb-16**	*Estimate*	4.157**	2.855***	1.240***
	SE	(1.493)	(0.774)	(0.341)
	95%CI	1.221 - 7.092	1.334 - 4.376	0.571 - 1.909
	p-value	(0.00563)	(0.000249)	(0.000288)
**Mar-16**	*Estimate*	4.410**	2.762***	1.298***
	SE	(1.608)	(0.794)	(0.369)
	95%CI	1.249 - 7.570	1.203 - 4.321	0.574 - 2.023
	p-value	(0.00637)	(0.000542)	(0.000457)
**Apr-16**	*Estimate*	4.047**	3.533***	1.445***
	SE	(1.404)	(0.728)	(0.387)
	95%CI	1.287 - 6.807	2.102 - 4.963	0.686 - 2.204
	p-value	(0.00416)	(1.61e-06)	(0.000198)
**May-16**	*Estimate*	4.665**	4.132***	2.221***
	SE	(1.658)	(0.865)	(0.483)
	95%CI	1.405 - 7.925	2.434 - 5.830	1.272 - 3.169
	p-value	(0.00515)	(2.26e-06)	(4.86e-06)
**Jun-16**	*Estimate*	4.735**	3.959***	2.348***
	SE	(1.608)	(0.797)	(0.425)
	95%CI	1.574 - 7.895	2.393 - 5.524	1.514 - 3.182
	p-value	(0.00341)	(9.15e-07)	(4.20e-08)
**Jul-16**	*Estimate*	4.670**	4.576***	2.762***
	SE	(1.528)	(0.882)	(0.503)
	95%CI	1.665 - 7.674	2.842 - 6.309	1.775 - 3.749
	p-value	(0.00240)	(3.03e-07)	(5.02e-08)
**Aug-16**	*Estimate*	5.889***	4.274***	2.340***
	SE	(1.760)	(0.813)	(0.453)
	95%CI	2.429 - 9.350	2.677 - 5.871	1.452 - 3.228
	p-value	(0.000898)	(2.09e-07)	(2.80e-07)
**Sep-16**	*Estimate*	4.911***	4.013***	2.686***
	SE	(1.436)	(0.796)	(0.474)
	95%CI	2.087 - 7.734	2.450 - 5.576	1.756 - 3.617

© 2023 Gracner T et al. *JAMA Network Open.*

Cont…		Mostly Public	Mixed	Mostly Private
**Outcome:**		***%FV of all WCV (if FV=0 in 2014)***	***%FV of all WCV (if FV=0 in 2014)***	***%FV of all WCV (if FV=0 in 2014)***
**Model:**		FE	FE	FE
	p-value	(0.000692)	(6.22e-07)	(1.92e-08)
**Oct-16**	*Estimate*	5.709**	4.338***	2.824***
	SE	(1.739)	(0.850)	(0.499)
	95%CI	2.290 - 9.127	2.668 - 6.008	1.844 - 3.804
	p-value	(0.00112)	(4.61e-07)	(2.00e-08)
**Nov-16**	*Estimate*	5.073***	5.502***	2.634***
	SE	(1.462)	(1.030)	(0.497)
	95%CI	2.198 - 7.947	3.479 - 7.525	1.659 - 3.609
	p-value	(0.000577)	(1.35e-07)	(1.40e-07)
**Dec-16**	*Estimate*	5.491**	5.505***	2.275***
	SE	(1.808)	(1.147)	(0.431)
	95%CI	1.937 - 9.044	3.251 - 7.758	1.429 - 3.121
	p-value	(0.00254)	(2.07e-06)	(1.60e-07)
**Jan-17**	*Estimate*	6.167***	6.214***	2.807***
	SE	(1.848)	(1.112)	(0.520)
	95%CI	2.534 - 9.800	4.029 - 8.398	1.787 - 3.827
	p-value	(0.000926)	(3.66e-08)	(8.23e-08)
**Feb-17**	*Estimate*	5.098**	6.267***	2.860***
	SE	(1.579)	(1.121)	(0.529)
	95%CI	1.994 - 8.201	4.065 - 8.468	1.821 - 3.898
	p-value	(0.00134)	(3.55e-08)	(8.14e-08)
**Mar-17**	*Estimate*	5.429**	6.553***	2.823***
	SE	(1.696)	(1.152)	(0.507)
	95%CI	2.095 - 8.764	4.291 - 8.815	1.828 - 3.818
	p-value	(0.00148)	(2.07e-08)	(3.29e-08)
**Apr-17**	*Estimate*	4.388***	7.187***	2.954***
	SE	(1.311)	(1.190)	(0.564)
	95%CI	1.810 - 6.966	4.851 - 9.524	1.848 - 4.061
	p-value	(0.000896)	(2.82e-09)	(1.96e-07)
**May-17**	*Estimate*	5.273**	6.474***	2.852***
	SE	(1.706)	(1.146)	(0.488)
	95%CI	1.919 - 8.627	4.224 - 8.724	1.895 - 3.808
	p-value	(0.00214)	(2.57e-08)	(6.62e-09)
**Jun-17**	*Estimate*	5.091**	6.779***	3.207***
	SE	(1.561)	(1.092)	(0.514)
	95%CI	2.023 - 8.159	4.633 - 8.924	2.199 - 4.216
	p-value	(0.00120)	(1.07e-09)	(6.34e-10)
**Jul-17**	*Estimate*	4.996***	7.639***	3.477***
	SE	(1.500)	(1.302)	(0.534)
	95%CI	2.048 - 7.944	5.080 - 10.20	2.429 - 4.526
	p-value	(0.000943)	(7.78e-09)	(1.20e-10)
**Aug-17**	*Estimate*	3.986**	7.851***	3.340***
	SE	(1.388)	(1.306)	(0.523)
	95%CI	1.258 - 6.714	5.287 - 10.42	2.313 - 4.367
	p-value	(0.00429)	(3.32e-09)	(2.66e-10)
**Sep-17**	*Estimate*	4.349***	7.422***	3.162***
	SE	(1.244)	(1.230)	(0.501)
	95%CI	1.904 - 6.793	5.005 - 9.838	2.179 - 4.145
	p-value	(0.000523)	(2.96e-09)	(4.11e-10)
**Oct-17**	*Estimate*	4.417***	6.829***	3.631***
	SE	(1.298)	(1.109)	(0.523)
	95%CI	1.865 - 6.969	4.650 - 9.008	2.605 - 4.657
	p-value	(0.000733)	(1.44e-09)	(0)
**Nov-17**	*Estimate*	4.056**	8.099***	4.479***
	SE	(1.288)	(1.268)	(0.594)
	95%CI	1.523 - 6.588	5.609 - 10.59	3.315 - 5.644
	p-value	(0.00177)	(3.56e-10)	(0)
**Dec-17**	*Estimate*	4.103**	8.007***	4.969***

© 2023 Gracner T et al. *JAMA Network Open.*

Cont…		Mostly Public	Mixed	Mostly Private
**Outcome:**		***%FV of all WCV (if FV=0 in 2014)***	***%FV of all WCV (if FV=0 in 2014)***	***%FV of all WCV (if FV=0 in 2014)***
**Model:**		FE	FE	FE
	SE	(1.415)	(1.383)	(0.657)
	95%CI	1.320 - 6.885	5.291 - 10.72	3.680 - 6.257
	p-value	(0.00395)	(1.18e-08)	(0)
**Year 2018**	*Estimate*	6.611***	10.02***	8.786***
	SE	(1.287)	(1.221)	(0.747)
	95%CI	4.081 - 9.141	7.623 - 12.42	7.320 - 10.25
	p-value	(4.37e-07)	(0)	(0)
No. observations		14,802	24,748	48,708

Notes: Standard errors are robust and clustered at the clinician level. */**/*** implies significantly different at p<0.05/0.01/0.001. Controls included were physician fixed effects, county-level measures of dentists per 1,000 population and pediatricians and family medicine physicians per 1,000 population <18 years old; a ZIP-code level measure of percentage of the population below 200% of the federal poverty level.

© 2023 Gracner T et al. *JAMA Network Open.*
