# Supplementary material for: The Patient Protection and Affordable Care Act and Pediatric Medical Clinicians’ Application of Fluoride Varnish
Source: JAMA Netw Open. 2023 Nov 14;6(11):e2343087. doi: 10.1001/jamanetworkopen.2023.43087 (PMC10646725; doi:10.1001/jamanetworkopen.2023.43087)
Supplement: Supplement 2. — Data Sharing Statement [file jamanetwopen-e2343087-s002.pdf]

Data Sharing Statement

Gracner. The Patient Protection and Affordable Care Act Mandate and Pediatric Medical Clinicians' Application of Fluoride Varnish. *JAMA Netw Open*. Published November 14, 2023. doi:10.1001/jamanetworkopen.2023.43087

Data
Data available: No

Additional Information
Explanation for why data not available: We cannot share our data, however others can purchase the data from CHIA (https://www.chiamass.gov) with approved DUA.
